# Go big or … don't? A field-based diet evaluation of freshwater piscivore and prey fish size relationships

**DOI:** 10.1371/journal.pone.0194092

**Published:** 2018-03-15

**Authors:** Jereme W. Gaeta, Tyler D. Ahrenstorff, James S. Diana, William W. Fetzer, Thomas S. Jones, Zach J. Lawson, Michael C. McInerny, Victor J. Santucci, M. Jake Vander Zanden

**Affiliations:** 1 Department of Watershed Sciences and the Ecology Center, Utah State University, Logan, Utah, United States of America; 2 Minnesota Department of Natural Resources, Brainerd, Minnesota, United States of America; 3 School of Natural Resources and the Environment, University of Michigan, Ann Arbor, Michigan, United States of America; 4 Wisconsin Department of Natural Resources, Madison, Wisconsin, United States of America; 5 Minnesota Department of Natural Resources, Aitkin, Minnesota, United States of America; 6 Wisconsin Department of Natural Resources, Mercer, Wisconsin, United States of America; 7 Minnesota Department of Natural Resources, Glenwood, Minnesota, United States of America; 8 Lake Michigan Program, Illinois Department of Natural Resources, Des Plaines, Illinois, United States of America; 9 Center for Limnology, University of Wisconsin – Madison, Madison, Wisconsin, United States of America; University of Hyogo, JAPAN

## Abstract

Body size governs predator-prey interactions, which in turn structure populations, communities, and food webs. Understanding predator-prey size relationships is valuable from a theoretical perspective, in basic research, and for management applications. However, predator-prey size data are limited and costly to acquire. We quantified predator-prey total length and mass relationships for several freshwater piscivorous taxa: crappie (*Pomoxis* spp.), largemouth bass (*Micropterus salmoides*), muskellunge (*Esox masquinongy*), northern pike (*Esox lucius*), rock bass (*Ambloplites rupestris*), smallmouth bass (*Micropterus dolomieu*), and walleye (*Sander vitreus*). The range of prey total lengths increased with predator total length. The median and maximum ingested prey total length varied with predator taxon and length, but generally ranged from 10–20% and 32–46% of predator total length, respectively. Predators tended to consume larger fusiform prey than laterally compressed prey. With the exception of large muskellunge, predators most commonly consumed prey between 16 and 73 mm. A sensitivity analysis indicated estimates can be very accurate at sample sizes greater than 1,000 diet items and fairly accurate at sample sizes greater than 100. However, sample sizes less than 50 should be evaluated with caution. Furthermore, median log_10_ predator-prey body mass ratios ranged from 1.9–2.5, nearly 50% lower than values previously reported for freshwater fishes. Managers, researchers, and modelers could use our findings as a tool for numerous predator-prey evaluations from stocking size optimization to individual-based bioenergetics analyses identifying prey size structure. To this end, we have developed a web-based user interface to maximize the utility of our models that can be found at www.LakeEcologyLab.org/pred_prey.

## Introduction

Body size is one of the most important aspects of animal and food web ecology [1–3]. An organism’s body size affects how it experiences the surrounding environment including energy needs, food availability, the amount of safely exploitable habitat, and predation risk [4, 5]. Consequently, size constraints govern predator-prey interactions [5], which in turn structure populations, communities, and food webs [6–8]. Understanding predator-prey size relationships is, therefore, critical to understanding food-web dynamics [8]. Furthermore, allometric relationships between predators and prey have been critical to numerous ecological theories such as ecological network theory [4], food web stability theory [9], and optimal foraging theory [10].

Predation plays a powerful role in structuring aquatic ecosystems [7] where predator-prey interactions among fishes are often size structured [11, 12]. Information on the minimum, median, and maximum prey sizes ingested by various piscivores is not only valuable from a theoretical perspective, but for a variety of basic research and management applications. These applications include identifying prey refuge sizes [11, 13], estimating potential vulnerability of native prey to nonnative predators [14, 15], determining appropriate stocking sizes to minimize predation [13, 15], and to minimizing assumptions in predator-prey modeling exercises (e.g., bioenergetics). However, minimum, median, and maximum ingested prey sizes are unknown or limited for many piscivorous fishes.

Information about size-specific vulnerability of prey fishes to piscivores is often limited to laboratory studies, gape-limit (i.e., predator mouth size) measurements, or field surveys using prey body depth rather than prey total length. Laboratory studies are problematic as they may not represent behavior or conditions observed in natural systems [16]. Gape-limit studies can provide information about theoretical upper limits of prey size, but lack information about predator foraging behavior. Indeed, natural selection drives predators to select prey that maximizes energy gain while minimizing search and handling times [17–19]. Consequently, predators in natural systems rarely forage at their gape-limit [20, 21], restricting the real-world applicability of gape-limit models. Therefore, information on sizes of prey fishes consumed by piscivores in natural ecosystems can not only bound the range of vulnerable prey sizes, but can also be used to identify the minimum, median, and maximum ingested prey fish sizes, as opposed to just a theoretical gape-limit.

Predator-prey fishes size relationships in freshwater ecosystems may also inform ecological theory. For instance, early optimal foraging theory, often used to predict piscivorous predator behavior, suggested that optimal prey size should increase linearly with predator size [10]. Subsequent research on marine fishes found that the relationship may not be linear as prey mobility can influence predation behavior and should be considered [21, 22]. However, predator-prey size relationships have been less studied in freshwater ecosystems. From a theoretical food web perspective, predator-prey body mass relationships are believed to govern stability in populations and food webs (i.e., popualiton or community persistence; [2, 9]). To date, however, information on predator-prey body mass relationships for freshwater fishes is limited, and the published accounts do not match theoretic predictions (see Discussion for more details; [5, 9]).

Here we compile numerous field-based diet datasets from several north temperate piscivorous fishes to evaluate the relationships between predator and prey total lengths and to quantify the minimum, median, and maximum ingested prey total lengths. When applicable, we identify differences in predator-prey total length relationships for fusiform and laterally compressed prey taxa as prey body depth may influence this relationship. We compare our findings to previous studies and discuss the shortcomings of various predator-prey total length relationship methods. We also use our data to evaluate model accuracy across sample size (i.e., how few predator-prey length observations can be obtained to still accurately estimate the minimum, median, and maximum ingested prey total lengths?). Finally, we use our robust dataset to evaluate predator-prey body mass relationship in the context of previous research investigating population and food web stability.

## Methods

Diet data for black crappie (*Pomoxis nigromaculatus*), largemouth bass (*Micropterus salmoides*), muskellunge (*Esox masquinongy*), northern pike (*Esox lucius*), rock bass (*Ambloplites rupestris*), smallmouth bass (*Micropterus dolomieu*), walleye (*Sander vitreus*), and white crappie (*Pomoxis annularis*) from lakes and reservoirs in Alberta, Illinois, Minnesota, New York, and Wisconsin were compiled from a variety of previously published and unpublished sources (Table 1; [23–28]). All diets were obtained via gastric lavage, the tube method, or dissection [29]. Predator total length and prey fish diet items were measured to the nearest mm, except for a subset of northern pike, which were measured to the nearest 5 mm. Prey species were identified to the lowest taxonomic level appropriate for the specific study. When prey fishes were too digested or incomplete to measure total length, total length was estimated using backbone to total length, fork length to total length, or standard length to total length relationships derived from the original dataset. When the original dataset was insufficient to derive study-specific length conversions (i.e., when only standard or fork lengths were reported without total length), we used length conversions from Carlander [30–32].

**Table 1:** Summary of compiled dataset sample sizes, location of lakes, and collection years.

Species	Predator sample size	Prey sample size	Lake Years	Lakes	State /Providence	Collection Years
Black Crappie	278	297	40	37	IL^1^, MN^2^	1988–2003
Largemouth Bass	959	1,486	29	22	IL^1^, MN^2^, NY^3^, WI^4^^,^^5^	1987–2013
Muskellunge	320	473	51	30	WI^6^^,^^7^	1991–2006
Northern Pike	784	2,233	7	2	Alberta^8^, MN^2^	1976–2013
Rock Bass	40	67	9	5	MN^2^, WI^9^	2001–2011
Smallmouth Bass	201	380	16	9	MN^2^, NY^3^, WI^5^^,^^9^	2001–2013
Walleye	5,375	18,102	21	8	IL^1^, MN^2^, WI^5^^,^^9^	1988–2013
White Crappie	14	20	9	9	MN^2^	2001–2003

Piscivores evaluated include black crappie (*Pomoxis nigromaculatus*), largemouth bass (*Micropterus salmoides*), muskellunge (*Esox masquinongy*), northern pike (*Esox lucius*), rock bass (*Ambloplites rupestris*), smallmouth bass (*Micropterus dolomieu*), walleye (*Sander vitreus*), and white crappie (*Pomoxis annularis*). Each year of study on a given lake was considered a unique lake year.

Data sources are as follows:

^1^Santucci and Wahl [28];

^2^MN DNR;

^3^ William (author of this study);

^4^Jereme Gaeta (author of this study);

^5^Craig Kelling, UW- Stevens Point;

^6^ Burri [25] and Bozek et al. [24];

^7^WI DNR;

^8^Diana [26];

^9^NTL-LTER [27]

Our goal was to quantify the relationship between prey and predator total lengths for each predator taxa. Specifically, we wanted to quantify the minimum, median, and maximum ingested prey total lengths. Preliminary analyses revealed predator-prey total length relationships were strongly non-homogeneous and not linear, which has been observed in other fish species (e.g., [21]). Consequently, standard linear regressions were inappropriate. To address the non-homogeneous variance and to generate model predictions of data extremes (e.g., the 99^th^ percentile regression), we used quantile regressions analyses [21, 33], which required linear data. Linearizing each predator-specific dataset to perform quantile regression analysis consisted of two steps: 1) we identified the best response variable (prey total length) transformation to meet the assumption of normality, and 2) we determined the predictor variable (predator total length) transformation that maximized linearity using Akaike Information Criterion (AIC) [34]. This procedure is detailed below.

We evaluated a suite of standard predator and prey total length transformations for each predator species to identify the best transformations to meet the assumptions of the quantile regression. We first assessed all combinations of prey total length^2^, total length, total length^1/2^, and log_e_ total length transformations to maximize normality based on a visual assessment of the transformed data and using a Shapiro-Wilk test for normality [35]. We then performed inverse probability weighted quantile regression analyses using the ‘quantreg’ (version 5.29) package in R Cran Statistical Software (version 3.0.2; [36]) on a suite of possible predator total length transformations (total length^2^, total length, total length^1/2^, and log_e_ total length). Imbalances across predictor variables (predator total length in this study) can limit applicability of model results at data extremes [37]. We, therefore, inverse probability weighted each observation using the predator species-specific Gaussian kernel density distribution to maximize the applicability of our models across the entire range of piscivore length [38]. In other words, each observation in our regression analyses was weighted by the inverse of the probability of a predator total length occurring in the dataset. That is, more common predator total lengths (i.e., the mode) being down-weighted and less common predator total lengths (i.e., the extremes) being up-weighted.

The best predictor variable transformation was determined via the AIC of the median (50^th^ percentile) quantile regression. We evaluated the 95^th^ percentile regression if median quantile regression AIC values were within 2 AIC units for two or more transformations [34]. In such cases, the transformation was cautiously identified using the 95^th^ percentile rather than the 99^th^ percentile as outliers may influence the 99^th^ percentile estimations, particularly when sample sizes are low [39]. When applicable, we repeated this procedure for laterally compressed and fusiform prey items (Table 2). The classification of taxa into prey shape can be found in S1 Appendix. To be conservative, we only report model results from the 5^th^ to 95^th^ percentile of predator total lengths for a given taxa and recommend that any future applications using our models are constrained to this length range as well.

**Table 2:** Predator and prey total length (TL, mm) transformations (trans.) and quantile regression derived equations.

Species	Prey shape	Predator trans.	Prey trans.	Equation
Crappie	All	$\sqrt{x}$	*log*_*e*_(*y*)	$Prey\;TL=exp(\alpha +\beta ∙\sqrt{predator\;TL})$
Largemouth Bass	All	*x*	*log*_*e*_(*y*)	*Prey TL* = *exp*(*α* + *β* ∙ *predator TL*)
	Fusiform	*log*_*e*_(*x*)	*log*_*e*_(*y*)	*Prey TL* = *exp*(*α* + *β* ∙ *log*_*e*_ (*predator TL*))
	Lat. comp.	*x*^2^	*log*_*e*_(*y*)	*Prey TL* = *exp*(*α* + *β* ∙*predator TL*^2^)
Muskellunge	All	*x*	*log*_*e*_(*y*)	*Prey TL* = *exp*(*α* + *β* ∙*predator TL*)
	Fusiform	*x*	*log*_*e*_(*y*)	*Prey TL* = *exp*(*α* + *β* ∙*predator TL*)
	Lat. comp.	*x*	$\sqrt{y}$	*Prey TL* = *exp*(*α* + *β* ∙*predator TL*)^2^
Northern Pike	All	*log*_*e*_(*x*)	*log*_*e*_(*y*)	*Prey TL* = *exp*(*α* + *β* ∙ *log*_*e*_ (*predator TL*))
Rock Bass	All	*x*^2^	*log*_*e*_(*y*)	*Prey TL* = *exp*(*α* + *β* ∙*predator TL*^2^)
Smallmouth Bass	All	*x*^2^	*log*_*e*_(*y*)	*Prey TL* = *exp*(*α* + *β* ∙*predator TL*^2^)
Walleye	All	*x*	*log*_*e*_(*y*)	*Prey TL* = *exp*(*α* + *β* ∙*predator TL*)
	Fusiform	*x*	*log*_*e*_(*y*)	*Prey TL* = *exp*(*α* + *β* ∙*predator TL*)
	Lat. comp.	*x*^2^	*log*_*e*_(*y*)	*Prey TL* = *exp*(*α* + *β* ∙*predator TL*^2^)

Piscivores evaluated include a grouped ‘crappie’ category (*P*. *nigromaculatus* and *P*. *annularis*), largemouth bass (*Micropterus salmoides*), muskellunge (*Esox masquinongy*), northern pike (*Esox lucius*), rock bass (*Ambloplites rupestris*), smallmouth bass (*Micropterus dolomieu*), and walleye (*Sander vitreus*). The analysis was performed on all prey shapes and, when sample sizes were sufficient, performed on fusiform and laterally compressed (lat. comp.) shaped prey as well.

In this study, we considered the 1^st^, 50^th^, and 99^th^ percentile regressions the minimum ingested prey total length (IP_min_), central prey total length tendency (IP_50_), and maximum ingested prey total length (IP_max_) observed in natural ecosystems (i.e., realized predator-prey length relationships), respectively. Sample sizes were large enough to prevent bias in the 99^th^ percentile regression for all species except rock bass (see Table A in S2 and S4 Appendices for rock bass results), for which we conservatively report the 95^th^ percentile regression [39]. The IP_50_ and IP_max_ prey total length categories were expressed as percentages of predator body length and are referred to as ‘relative IP_50_’ and ‘relative IP_max_’, respectively. We also performed a literature review of previously reported gape-limits and maximum observed prey sizes to place our results in the context of previous research.

Distributions of prey total lengths at specific predator total lengths were estimated using Gaussian kernel densities of quantile regression estimates for every percentile from the 1^st^ to the 99^th^ percentile. Prey length distributions were calculated for the 5^th^, 50^th^, and 95^th^ percentile of predator total lengths. We tested for differences among these distributions using non-parametric multiple comparisons with the ‘pgirmess’ package (version 1.6.7) [40].

Previous predator-prey total length research has restricted IP_max_ to the 95^th^ or even 90^th^ percentile regression based on sample size [39, 41]. We, therefore, evaluated the sensitivity of IP_min_, IP_50_, and IP_max_ to sample size. To this end, we resampled our largest dataset (walleye; n = 18,102) without replacement to generate new predator-prey total length datasets at reduced sample sizes ranging from n = 1,000 to n = 5. The 1^st^, 50^th^, and 99^th^ percentile regressions (IP_min_, IP_50_, and IP_max_) were analyzed at each reduced sample size. Model-estimated prey total length was calculated for the 1^st^ 50^th^, and 99^th^ percentile of predator total lengths observed in the full dataset (131, 483, and 682 mm, respectively). Prey length estimates at reduced sample sizes were compared to the estimates derived with the full dataset to determine the deviation from the full sample size as a percent difference. This procedure was repeated 1,000 times at each sample size to evaluate variance.

While the primary objective of our work was to evaluate predatory-prey length relationships, predator-prey body mass relationships are critical to understanding population and food web dynamics and stability [2, 3, 9, 42]. Therefore, we compared our predator-prey observations to the large dataset compiled by Brose et al. [43] and used in Brose et al. [9] to place our observations in the context of population and food web stability. We converted our predator and prey fish length data to mass with the following allometric relationship used in Brose et al. [43]:

$${\text{mass}=10,600(\text{length})}^{2.57}$$

where the units of fish length and mass are meters and grams, respectively. We compared our taxa-specific observations to those of Brose et al. [43]. Specifically, we used the same freshwater (stream and lake combined) ectotherm predator-prey body mass relationship documented in Brose et al. [42] and further reduced predator types from all freshwater ectotherms to just freshwater fishes (i.e., removing non-fish ectotherms such as frogs and snakes) and also just freshwater piscivores (i.e., only observations of fish eating other fishes). More specifically, we took an individual-link predator-prey body mass ratio approach [3, 44], as recommended by Nakazawa et al. [3], with body mass ratios calculated as follows:

$$\frac{\text{Mass}\;\text{of}\;\text{the}\;\text{individual}\;\text{predator}}{\text{Mass}\;\text{of}\;\text{an}\;\text{individual}\;\text{prey}\;\text{item}\;\text{consumed}\;\text{by}\;\text{the}\;\text{individual}\;\text{predator}}$$

We compared these data using an ANOVA followed by a Tukey HSD test with the Bonferroni corrected significance at the *p* ≤ 0.05 level.

## Results

**Fig 1:**
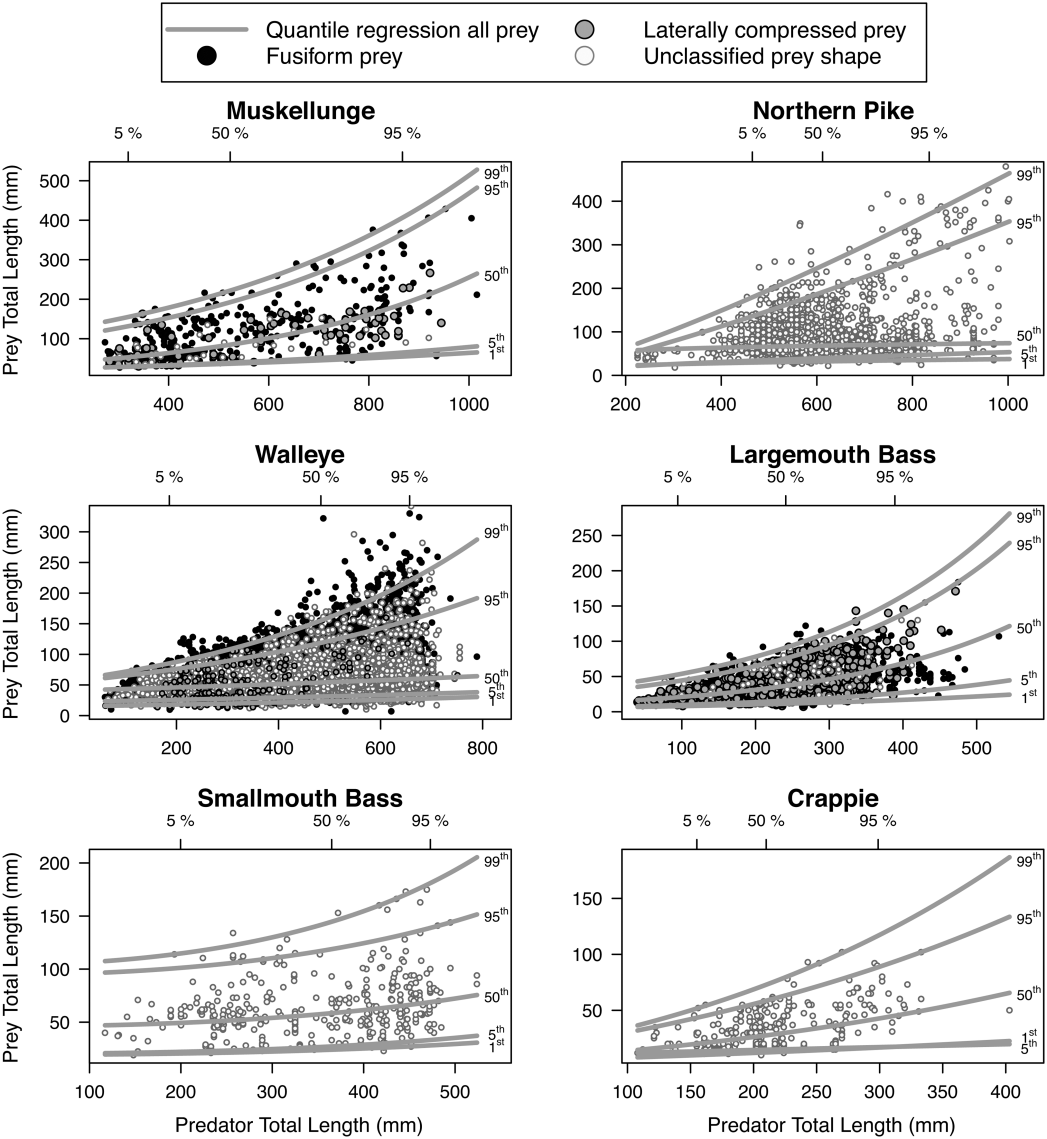
Predator and prey fish total lengths (mm) and quantile regression models. Piscivores evaluated include muskellunge (*Esox masquinongy*; n = 473), northern pike (*Esox lucius*; n = 2,233), walleye (*Sander vitreus*; n = 18,102), largemouth bass (*Micropterus salmoides*; n = 1,486), smallmouth bass (*Micropterus dolomieu*; n = 380), and a grouped ‘crappie’ category (*P*. *nigromaculatus* and *P*. *annularis*; n = 317). The 1^st^, 5^th^, 50^th^, 95^th^, and 99^th^ percentile regressions are shown as gray lines. When the appropriate taxonomic resolution and sample size was available, prey fishes were categorized as having fusiform (black points) or laterally compressed (gray points) body shape, otherwise prey fish body shape was unclassified (open points). The 5^th^, 50^th^, and 95^th^ percentiles of predator total lengths are shown at the top of each plot and correspond to the range of lengths modeled in Figs 2 and 3 as well as the density distributions in Fig 4.

The range of prey total lengths consumed increased with predator total length for all predator taxa (Fig 1). The IP_50_ (50^th^ percentile regression) increased by >200% between the 5^th^ and 95^th^ percentiles of predator total length (see Fig 1 top axes) for muskellunge and largemouth bass, by 112% for crappie, but remained fairly constant, only increasing by 40% to 53% from the 5^th^ to 95^th^ percentiles of predator total length, for northern pike, walleye, and smallmouth bass (Fig 1, Table 3). The IP_max_ (99^th^ percentile regression) increased by more than 126% across the 5^th^ and 95^th^ percentiles of predator total length for muskellunge, northern pike, walleye, largemouth bass, and crappie, but only increased by 58% for smallmouth bass. While the IP_min_ (1^st^ percentile regression) from the 5^th^ to the 95^th^ percentiles of predator total length increased by between 37% and 108% for all taxa, this accounted for an increase of only 7–14 mm for all taxa except for muskellunge, which increased by 26 mm.

**Table 3:** IP_50_ (50^th^ percentile regression) and IP_max_ (99^th^ percentile regression) model coefficients.

Species	Prey shape	Percentile	Intercept (*α*)	Coefficient (*β*)
Crappie	All	50^th^	1.07e-00	1.55e-01
	All	99^th^	1.85e-00	1.69e-01
Largemouth Bass	All	50^th^	2.65e-00	3.94e-03
	All	99^th^	3.61e-00	3.72e-03
	Fusiform	50^th^	1.55e-00	4.25e-01
	Fusiform	99^th^	4.43e-01	7.74e-01
	Lat. comp.	50^th^	2.83e-00	1.04e-05
	Lat. comp.	99^th^	3.93e-00	7.05e-06
Muskellunge	All	50^th^	3.23e-00	2.31e-03
	All	99^th^	4.48e-00	1.76e-03
	Fusiform	50^th^	3.52e-00	1.98e-03
	Fusiform	99^th^	4.57e-00	1.61e-03
	Lat. comp.	50^th^	3.57e-00	1.05e-02
	Lat. comp.	99^th^	1.06e+01	6.23e-03
Northern Pike	All	50^th^	1.62e-00	4.13e-01
	All	99^th^	-3.70e-00	1.43e-00
Rock Bass	All	50^th^	3.23e-00	1.64e-05
	All	95^th^	4.13e-00	1.13e-05
Smallmouth Bass	All	50^th^	3.83e-00	1.82e-06
	All	99^th^	4.64e-00	2.48e-06
Walleye	All	50^th^	3.59e-00	9.06e-04
	All	99^th^	4.07e-00	2.00e-03
	Fusiform	50^th^	3.62e-00	9.24e-04
	Fusiform	99^th^	4.12e-00	1.97e-03
	Lat. comp.	50^th^	3.18e-00	3.73e-06
	Lat. comp.	99^th^	4.51e-00	1.52e-06

Piscivores evaluated include a grouped ‘crappie’ category (*P*. *nigromaculatus* and *P*. *annularis*), largemouth bass (*Micropterus salmoides*), muskellunge (*Esox masquinongy*), northern pike (*Esox lucius*), rock bass (*Ambloplites rupestris*), smallmouth bass (*Micropterus dolomieu*), and walleye (*Sander vitreus*). The analysis was performed on all prey shapes and, when data was sufficient, performed on just fusiform and laterally compressed (lat. comp.) shaped prey. Coefficients correspond to those in Table 2. Coefficients values of the 50^th^ and 99^th^ percentile regressions correspond to those in Fig 1 and are shown for all species except Rock Bass for which we report the 95^th^ percentile due to low sample size. Every percentile regression coefficients from the 1^st^ to the 99^th^ for all predator fishes and prey shapes can be found in S2 Appendix.

**Fig 2:**
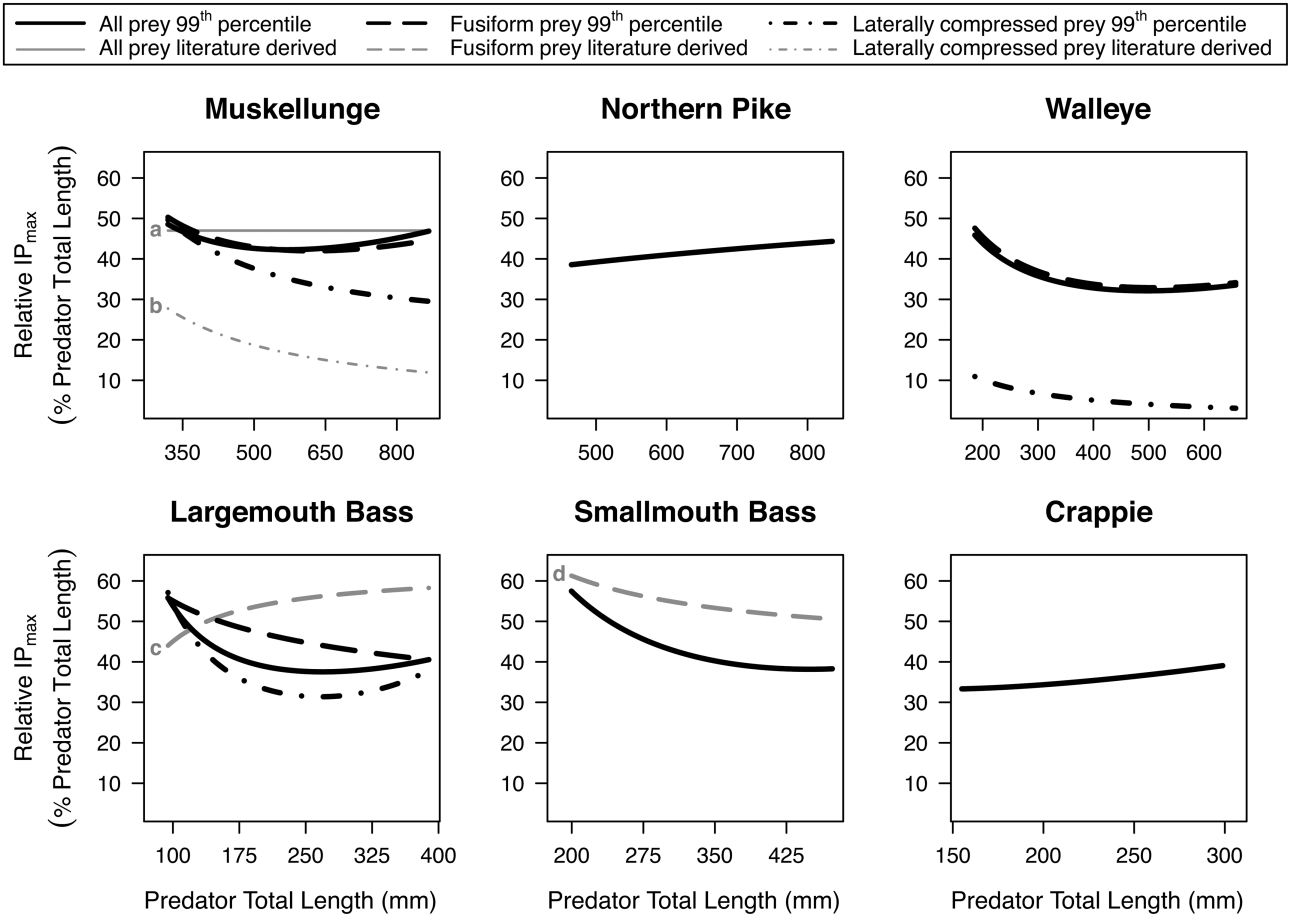
The relative maximum ingested prey total length (percent of predator total length) consumed across predator total length. Piscivores evaluated include muskellunge (*Esox masquinongy*), northern pike (*Esox lucius*), walleye (*Sander vitreus*), largemouth bass (*Micropterus salmoides*), smallmouth bass (*Micropterus dolomieu*), and a grouped ‘crappie’ category (*P*. *nigromaculatus* and *P*. *annularis*). The relative IP_max_ (99^th^ percentile regression) is shown from the 5^th^ to 95^th^ percentile of observed predator total lengths, which are noted on the top axes of Fig 1. When applicable, we estimated relative IP_max_ for different prey body shapes: fusiform (dashed lines) and laterally compressed (dotted lines). Literature derived data are for ^a^ field survey of all prey [24]; ^b^ field survey of gizzard shad (*Dorosoma cepedianum*) as prey [45], ^c^ gape-limit for largemouth bass as prey [46], and ^d^ field survey of Cyprinids as prey [14]. Additional literature derived estimates are reported in S3 Appendix.

**Fig 3:**
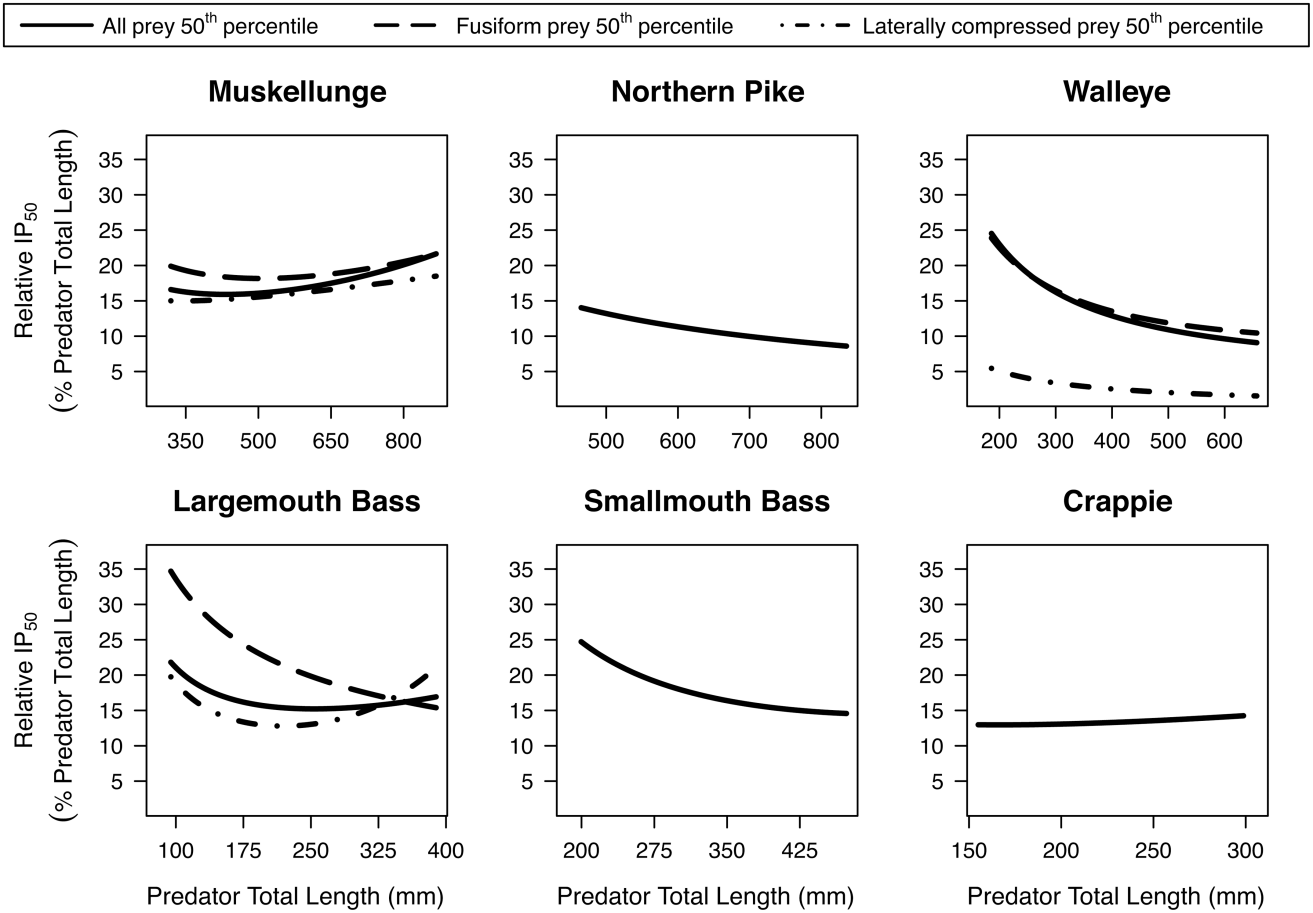
Central tendency of relative ingested prey total length (percent of predator total length) consumed across predator total length. Piscivores evaluated include muskellunge (*Esox masquinongy*), northern pike (*Esox lucius*), walleye (*Sander vitreus*), largemouth bass (*Micropterus salmoides*), smallmouth bass (*Micropterus dolomieu*), and a grouped ‘crappie’ category (*P*. *nigromaculatus* and *P*. *annularis*). The relative IP_50_ (50^th^ percentile regression) is shown from the 5^th^ to 95^th^ percentile of observed predator total lengths, which are noted on the top axes of Fig 1. When applicable, we estimated relative IP_50_ for different prey body shapes: fusiform (dashed lines) and laterally compressed (dotted lines).

Relative IP_max_ (IP_max_ as a percentage of predator total length) was fairly constant across predator total length for muskellunge and northern pike; decreased with predator total length for walleye, largemouth bass, and smallmouth bass; and increased slightly for crappie (Fig 2). Predators tended to have lower IP_max_ for laterally compressed prey compared to fusiform prey. Across all taxa, relative IP_max_ for the 5^th^ to 95^th^ percentile range of predator total lengths ranged from 32–57% with the interquartile range (i.e., the middle 50%) of these estimates spanning from 32–46%. When prey shape was accounted for, the interquartile range of the estimates of relative IP_max_ ranged from 33–49% for fusiform prey items and from 4–39% for laterally compressed prey total lengths. The relative IP_50_ of our estimates based on all diet data across all taxa ranged from 9–25%, with the interquartile range spanning from 10–20% (Fig 3). When prey shape was accounted for, the interquartile range of the relative IP_50_ estimates ranged from 11–25% for fusiform prey items and from 2–17% for laterally compressed prey total lengths. Generally, the relative IP_50_ increased across length for muskellunge, largemouth bass and crappie, but tended to decrease or remain constant for all other predator taxa.

**Fig 4:**
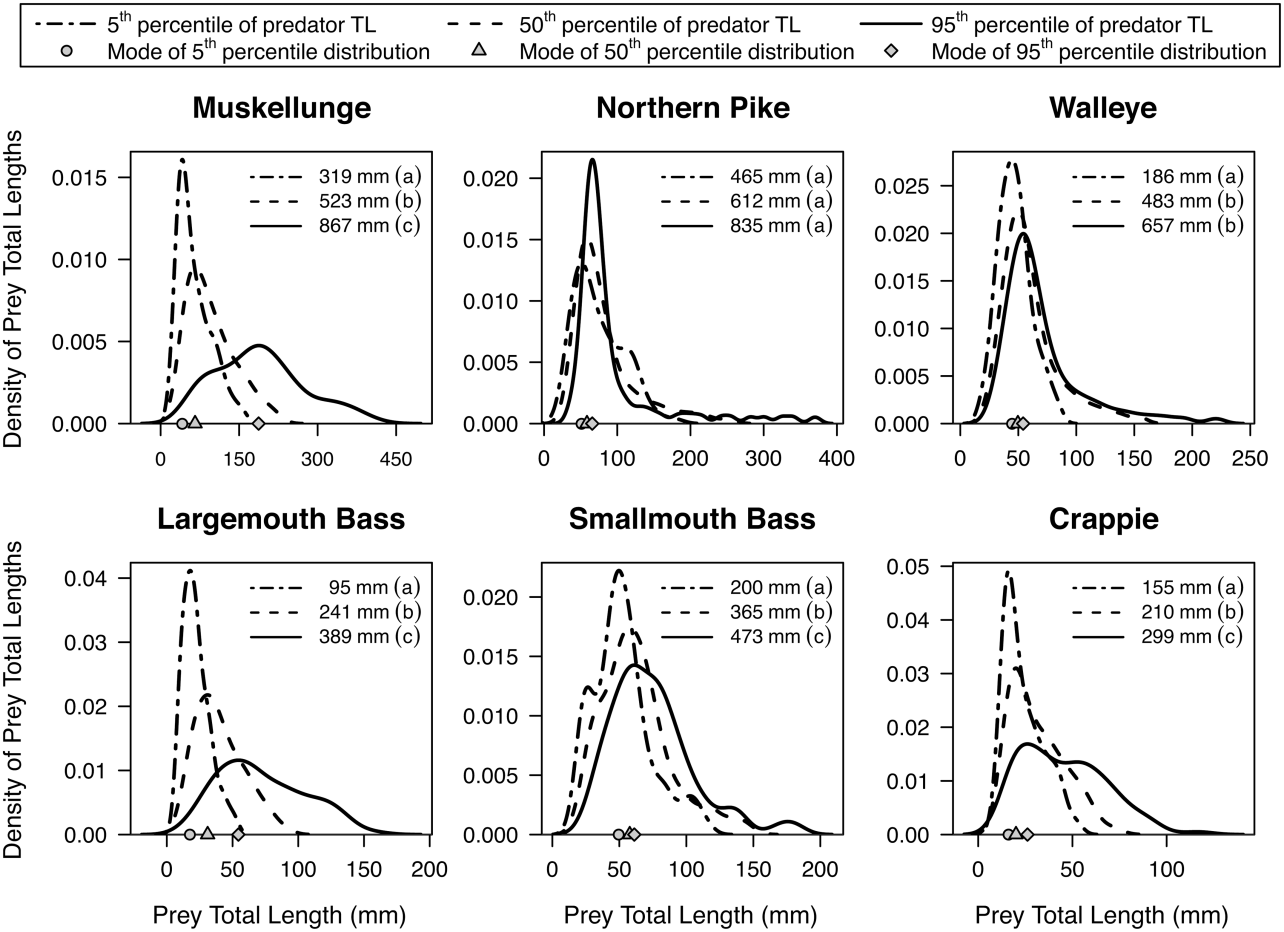
Kernel density distributions of model estimated consumed prey total lengths (mm). Distributions estimated for the 5^th^ (dotted line), 50^th^ (dashed line), and 95^th^ (solid line) percentile of predator total lengths (TL; mm). Piscivores evaluated include muskellunge (*Esox masquinongy*), northern pike (*Esox lucius*), walleye (*Sander vitreus*), largemouth bass (*Micropterus salmoides*), smallmouth bass (*Micropterus dolomieu*), and a grouped ‘crappie’ category (*P*. *nigromaculatus* and *P*. *annularis*). The predator total lengths correspond to the top axes of Fig 1. Kernel densities at a given predator total length were derived by estimating prey total length with percentile regressions of every percentile from the 1^st^ to the 99^th^ (Table A in S2 Appendix). The modes of the 5^th^, 50^th^, and 95^th^ percentile kernel density distributions are shown along the bottom axis as gray circles, triangles, and diamonds, respectively.

Estimated kernel distributions of prey total length for the 5^th^, 50^th^, and 95^th^ percentiles of observed predator total lengths indicated that the prey total length distributions shifted toward slightly larger prey items with increasing predator total length (Fig 4). Muskellunge showed the greatest increase in prey total length with predator total length, followed by largemouth bass. For all other predator taxa, however, prey total length distributions had nearly identical modes for the 5^th^, 50^th^, and 95^th^ percentiles of observed predator total lengths. Indeed, with the exception of the 95^th^ percentile of muskellunge total length exhibiting a modal prey total length of 187 mm, the modal prey total lengths consistently fell within the relatively narrow range of 16 to 73 mm, regardless of predator taxa or length.

**Fig 5:**
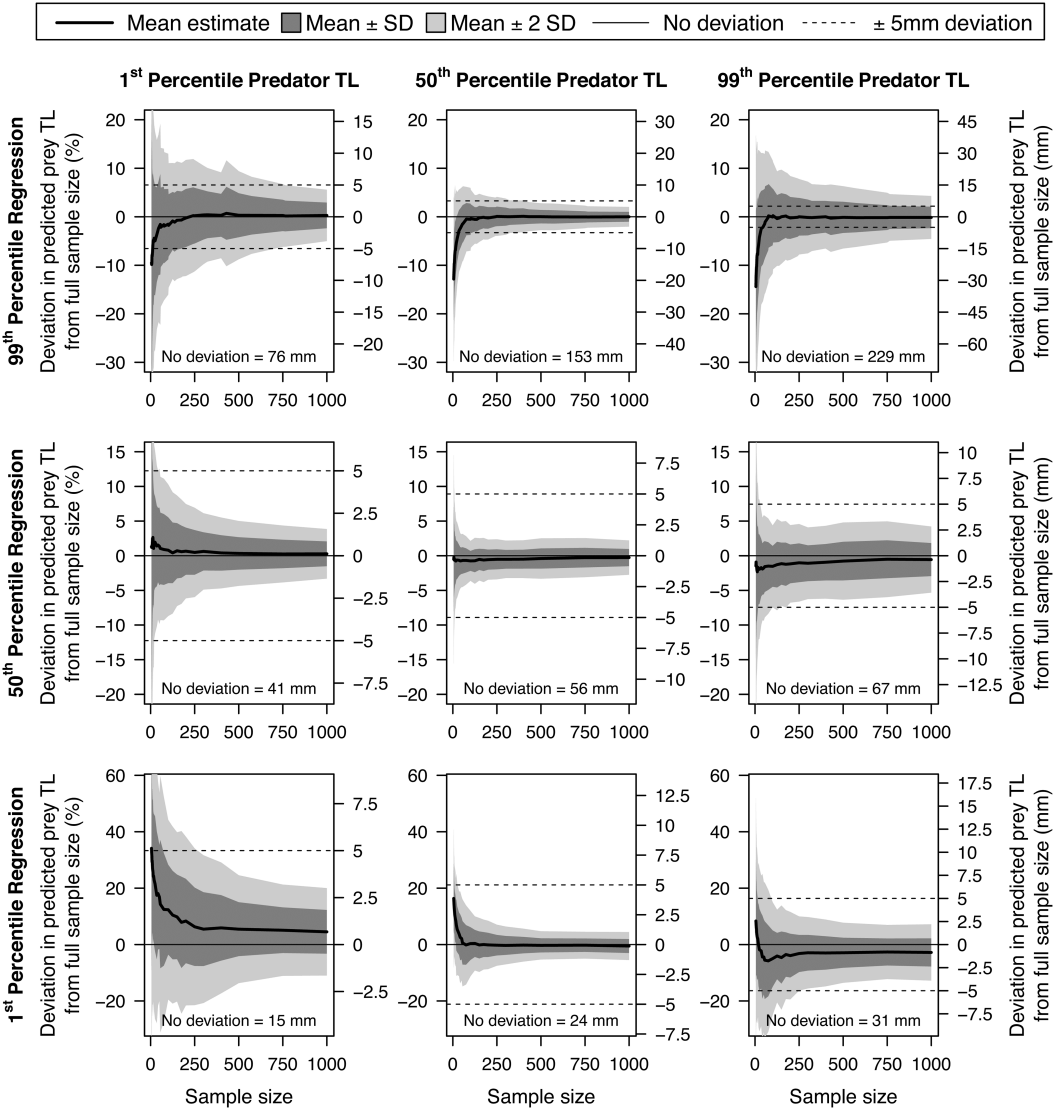
The deviation in predicted prey total length (TL) across sample size. We resampled our largest dataset (walleye; n = 18,102) without replacement generating a range of smaller sample sizes and evaluated how sensitive prey total length predictions are to the number of observations used to develop the model. The 1^st^, 50^th^, and 99^th^ percentile regression (i.e., the minimum, median, and maximum ingested prey total length models; IP_min_, IP_50_, and IP_max_, respectively) was reanalyzed at each reduced sample size and the predicted prey total length consumed at the 1^st^, 50^th^, 99^th^ percentiles of predator total length (131, 483, and 682 mm, respectively) was estimated. These estimates were compared to the model-estimated prey total length derived with the full dataset (n = 18,102) to determine the deviation as sample size is reduced. This procedure was repeated 1,000 times at each sample size to calculate the mean deviance (thick black line), ± 1 standard deviation (SD; dark gray polygon), and ± 2 standard deviations (light gray polygon). Shown with ± 5 mm as thin dashed lines.

The sensitivity of the IP_min_, IP_50_, and IP_max_ estimated prey total length to sample size was minimal for sample sizes greater than 1,000 observations and, while the variance increased, the mean estimate was always within 5 mm of the model estimate derived from the full sample size until sample sizes fell well below 50 observations (Fig 5). More specifically, the model predicted IP_min_, IP_50_, and IP_max_ at the most extreme predator total lengths (the 1^st^ and 99^th^ percentile of walleye length in the full data set, which should be the most sensitive to reductions in sample size; 131 and 682 mm, respectively) differed from the full sample by <4.5% (σ = ±<7.8%) or <1mm (σ = ±<1.5 mm), <0.6% (σ = ±<2.4%) or <1mm (σ = ±<1.6 mm), and <0.3% (σ = ±<2.7%) or <1mm (σ = ±<5.1 mm), respectively, for sample sizes greater than 1,000. At sample sizes of greater than 100 observations, the mean model predicted IP_min_, IP_50_, and IP_max_ remained within 12.4% or <2mm, 1.5% or 1mm, and 1.8% or <2mm of the full sample size derived model, respectively; however, the standard deviation increases to ±15.9% or ±3 mm, ±4.8% or ±2 mm, and ±7.3% or ±13 mm, respectively. While the mean IP_min_, IP_50_, and IP_max_ observation remained within 3 mm, 1 mm, and 5 mm of the full sample size derived model to sample sizes as low as 50 observations, respectively, the variance increased sharply at low sample sizes (i.e., ±18.4% or ±4 mm, ±5.6% or ±3 mm, and ±10.3% or ±16 mm).

**Fig 6:**
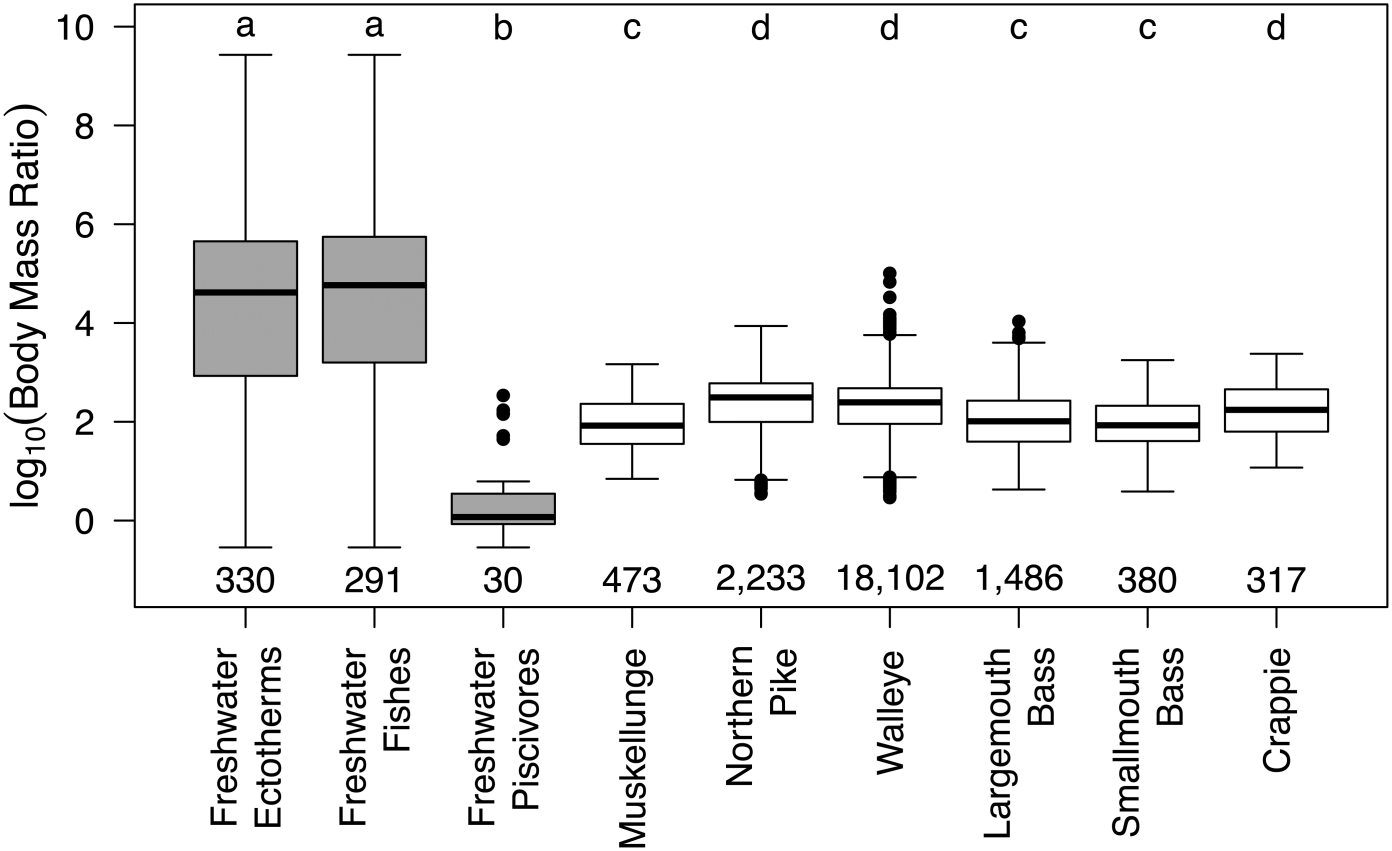
Predator-prey body-mass ratios (log_10_) across predator and prey taxa. Gray boxes are data from Brose et al. [43] with "Freshwater ectotherms" reported in Brose et al. [42]; the data were further subsetted into only freshwater fish as predators ("Freshwater fishes") and freshwater fishes preying on fishes ("Freshwater piscivores"). White boxes represent data from our study. Shown with sample size (below); groups with the same letter are not significantly different at the p≤0.05 level. Box plots are shown with medians, first and third quartiles, and a range of 1.5 times the interquartile range. Outliers beyond the range are represented as points. Fish lengths were converted into mass using the general allometric relationship for fishes as reported in Brose et al. [43].

Predator-prey body mass ratios of piscivorous taxa in our study were significantly different from the freshwater ectotherms, fishes, and piscivores reported in Brose et al. [42, 43] (Fig 6). We found that the median individual-link log_10_ body mass ratio for freshwater ectotherms and fishes evaluated by Brose et al. [42, 43] were 4.6 and 4.8, respectively, while the ratio was <0.1 for freshwater piscivorous fishes from their study. Conversely, the median individual-link log_10_ body mass ratio among our piscivorous taxa ranged from 1.9 to 2.5.

## Discussion

We observed non-linear predator-prey total length relationships as the range of prey total lengths increased with predator total length across all taxa (Figs 1 and 4). Overall, this resulted in larger individuals of a given taxa consuming a larger range of prey total lengths relative to smaller individuals, a phenomenon known to occur across ecosystems, taxa, and trophic levels [1, 47, 48]. Our findings also support previous research suggesting that piscivores generally consume relatively small prey regardless of predator total length [21, 22, 48, 49], which may not reflect size structure of prey fishes available in the ecosystem [50]. While the range of prey total lengths increased with predator total length for all piscivores studied here (Fig 1), we found two distinct patterns in predator-prey total length relationships (Fig 4):

1. Predators tend to always consume small prey (e.g., Fig 1 walleye). This is illustrated by a constant central prey total length tendency (IP_50_) with predator total length that is much closer to the minimum ingested prey total length (IP_min_) than the maximum (IP_max_) [49]. In other words, the relative IP_50_ decreases with predator total length (e.g., Fig 3 walleye).
2. Predators consume increasingly larger prey with predator total length. This is characterized by the IP_50_ increasing with predator total length at a similar rate as the IP_max_ (Fig 1 muskellunge). That is, the relative IP_50_ increases or is relatively constant across predator total length (e.g., Fig 3 muskellunge).

The former pattern is commonly reported in the literature for piscivores (e.g., see review by Juanes [49]). In our study, the former pattern held for northern pike, smallmouth bass, and walleye. However, we observed the latter pattern in crappie, largemouth bass, muskellunge, and rock bass, a pattern previously reported only for largemouth bass [51].

The most common (modal) prey total length for all taxa studied here, with the exception of large muskellunge, ranged from 16–73 mm. This suggests that piscivores are preying on commonly available small prey fish sizes that are likely easier to catch than larger prey [50], and that small fishes (16–73 mm) may be the optimal prey total length maximizing energy intake per unit time regardless of piscivore length or taxa [2]. Indeed, our findings of a non-linear increase in prey total length with predator total length gives credence research concluding that optimal foraging on mobile prey is not solely a function of maximizing energy gain while minimizing handling time, but may be driven by capture success [48, 52–54].

Early optimal foraging (or diet) theory predicted that predators optimally forage by selecting prey that maximizes energy gain while minimizing handling time [2, 10, 17–19]. Optimal foraging theory, therefore, predicts that IP_max_ and IP_50_ should increase linearly with predator size [10] and is limited only by gape (i.e., gape-limit; [21]). While this has served as a foundation for gape-limitation research, studies over the last quarter century have shown that piscivore predation does not follow this pattern as prey mobility may influence both encounter rate and capture efficiencies [21, 22, 48, 52–54]. Furthermore, energetically favorable large prey fishes are often relatively scarce in ecosystems [48]. While piscivores become more effective predators with size due to increased swimming speed, burst capabilities, and visual acuity, prey fishes similarly become more effective at avoiding predation with size [2, 48, 55]. Our findings support these developments in optimal foraging theory that suggest foraging success on mobile prey is not simply a function of gape limitation and handling time, but also of search time, encounter rate, opportunity, and prey behavior [52, 53].

### Limits and shortcomings of estimates of predator-prey total length relationships

Predator-prey total length relationships are estimates and must be considered within the context of the data used to derive the models. Critical context-specific characteristics of the data should be considered including the type of study (i.e., field or laboratory), the type of measurement (i.e., diet observations or gape-size), the sample size (i.e., the number of ecosystems, the number of prey items, and the number of predators in the study), the shape of the prey taxa (i.e., fusiform or laterally compressed), and how the predator-prey relationship was derived (i.e., regression-based or single point estimate). For instance, we found that single point estimates such as the single maximum or the 90^th^ percentile predator-prey total length observed in a dataset, fail to capture non-linear patterns across predator total length (Fig 2, Line a; Table 4; e.g., [24]). Likewise, using data from only one system may bias the predator-prey total length relationships, as a full range of possible prey total lengths may not be available to predators (Fig 2, Line b; Table 4; e.g., [45]).

**Table 4:** Literature review of study piscivore gape-limits and maximum ingested prey size estimates.

Predator taxon	Prey taxon	Study Type	Estimate Type	% Body Length	Reference
Crappie	All Prey	Field survey	90^th^ %tile value	32%	Pierce, Sexton [57]
Crappie	All Prey	Field survey	Max. value	50%	Pierce, Sexton [57]
Largemouth Bass	All Prey	Field survey	Max. model	30–35%	Goldstein [58]
Largemouth Bass	All Prey	Field survey	90^th^ %tile value	33%	Pierce, Sexton [57]
Largemouth Bass	All Prey	Field survey	Max. value	71%	Pierce, Sexton [57]
Largemouth Bass	Bluegill	Gape-limit	Max. model	34–35%	Lawrence [56]
Largemouth Bass	Gizzard Shad	Gape-limit	Max. model	34–49%	Lawrence [56]
Largemouth Bass	Largemouth Bass	Gape-limit	Max. model	44–58%	Lawrence [56]^c^
Muskellunge	All prey	Field survey	Max. value	47%	Bozek, Burri [24]^a^
Muskellunge	Bluegill	Field survey	Max. model	13–20%	Wahl and Stein [45]
Muskellunge	Gizzard Shad	Field survey	Max. model	12–28%	Wahl and Stein [45]^b^
Northern Pike	All Prey	Field survey	90^th^ %tile value	28%	Pierce, Sexton [57]
Northern Pike	All Prey	Field survey	Max. value	50%	Pierce, Sexton [57]
Northern Pike	Bluegill	Field survey	Max. model	20–23%	Wahl and Stein [45]
Northern Pike	Gizzard Shad	Field survey	Max. model	40–42%	Wahl and Stein [45]
Smallmouth Bass	All Prey	Field survey	90^th^ %tile value	35%	Pierce, Sexton [57]
Smallmouth Bass	All Prey	Field survey	Max. value	78%	Pierce, Sexton [57]
Smallmouth Bass	Cottidae	Field survey	Max. model	38–53%	Zimmerman [14]
Smallmouth Bass	Cyprinidae	Field survey	Max. model	51–61%	Zimmerman [14]^d^
Smallmouth Bass	Salmonidae	Field survey	Max. model	32–51%	Zimmerman [14]
Walleye	All Prey	Field survey	Max. model	37–43%	Parsons [59]
Walleye	All Prey	Field survey	Max. model	24–51%	Knight, Margraf [60]
Walleye	All Prey	Field survey	90^th^ %tile value	32%	Pierce, Sexton [57]
Walleye	All Prey	Field survey	Max. value	56%	Pierce, Sexton [57]
Walleye	Cyprinidae	Field survey	Max. model	27–36%	Zimmerman [14]

Studies are classified as either field surveys or studies measuring predator gape (i.e., gape-limit). The estimate is classified as a continuous maximum model (max. model), a single maximum value (i.e, 100^th^ percentile; max. value), or a 90^th^ percentile value (90^th^ %-tile).

^a—d^ correspond to literature derived estimates shown in Fig 2.

A common approach to estimate IP_max_ is the gape-limit method (Fig 2, Line c; Table 4; e.g., [56]). However, this method assumes predator mouth size is the only determinant of prey size and does not account for prey availability, prey behavior, handling time, capture success, or competition, which often results in overestimated IP_max_ for larger individuals [20, 21, 54]. Another important factor to consider is that predators may behave very differently in novel systems such as experimental tanks or in an invaded ecosystem. For instance, we observed a lower IP_max_ for smallmouth bass than that observed by a previous survey studying this species in its invasive range (Fig 2, Line d; Fig A in S3 Appendix; [14]). We also found that prey shape (i.e., fusiform or laterally compressed) may drastically affect the estimated predator-prey total length relationship, with predators often consuming smaller laterally compressed fishes compared to fusiform ones (Figs 2 and 3).

We acknowledge that our results have limitations (e.g., low sample sizes for crappie, smallmouth bass, and rock bass; northern pike observations from only two lakes; and a lack of information on prey fish community size structure available in the ecosystem) and, therefore, stress that these are “realized” prey lengths, not “preferred” prey lengths. Additionally, we recommend that future implementations of our models conservatively limit applications to between the 5^th^ and 95^th^ percentiles of predator total length observed in our study as unusual patterns can occur beyond these ranges (e.g., crossing of the 1^st^ and 5^th^ percentile regressions for crappie; Fig 1). Furthermore, while it has been shown that the size distribution of prey fishes available in the environment do not reflect those observed in the diet [50], we recommend future analyses compare the distribution of prey total lengths found in diets to the distribution of length observed in the ecosystem. Despite potential shortcomings, examining predator-prey total length relationships for a variety of taxa across multiple lakes, as in our study, provides an empirical basis for assessing how predation can structure or influence populations, communities, and aquatic ecosystems.

### Predator-prey body mass ratios of freshwater fishes

In the largest analysis of predator-prey body mass ratios across ecosystems (i.e., terrestrial, marine, freshwater) and predator types (invertebrate, ectothermic vertebrate, and endothermic vertebrate), Brose et al. [42] found log_10_ body mass ratios for freshwater vertebrates of approximately 4, which means that predators were 10,000 times larger than their prey. However, they acknowledged a major shortcoming of their study being freshwater samples based largely on fishes consuming only invertebrates rather than piscivorous fishes. In the same study, terrestrial and marine log_10_ predator-prey body mass ratios were closer to 2. Furthermore, Brose et al. [9], assessed population and food web stability using theoretical models (structural food web and non-linear bioenergetics models, specifically), and found that ectothermic vertebrate populations should be most stable and food webs should begin to stabilize when ectothermic vertebrate predator-prey log_10_ body mass ratios are around 2 (i.e., predator mass being 100 times larger than prey). While our data are inherently biased in the opposite way of Brose et al. [42] by including only piscivory, our findings, in conjunction with Brose et al. [42], support the theoretical conclusions of Brose et al. [9], suggesting that the optimal predator-prey log_10_ body mass ratio for all ectothermic vertebrates, regardless of ecosystem, is approximately 2 to 3. An important caveat of our work is that our log_10_ body mass ratios are “realized” (i.e., observed in a diet and does not take environmental variability of prey size availability into account) and not “preferred” (i.e., the predator selection given the availability of all possible prey sizes) [61]. As noted by Brose et al. [42], additional data and further research is needed to assess differences among pelagic and benthic predator-prey mass ratios for both freshwater and marine ecosystems.

### Case study application

**Fig 7:**
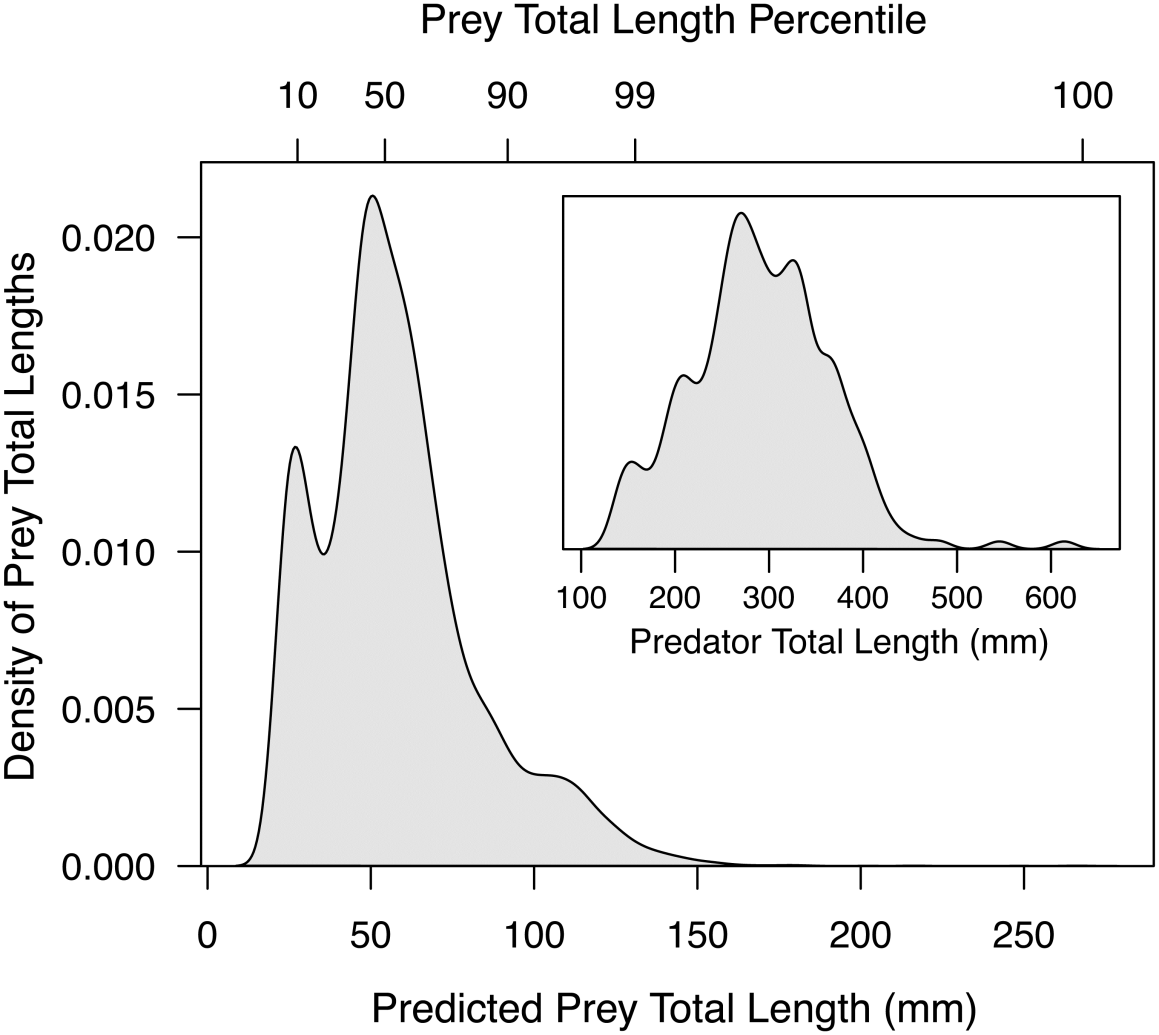
A management application minimizing vulnerability of stocked prey fish to predation in a Utah reservoir. The kernel density distribution of model predicted prey total lengths (mm) consumed by a smallmouth bass population (inset) in Big Sand Wash Reservoir, UT. Shown with percentiles of the prey total lengths consumed on the top axis.

Managers in the Wisconsin Department of Natural Resources and the Utah Division of Wildlife Resources (UT-DWR) have already used the models presented here to inform management actions. For example, UT-DWR managers are implementing a triploid walleye stocking program in Big Sand Wash Reservoir, UT to combat a walleye invasion. However, the reservoir contains a large smallmouth bass population that is likely to heavily prey upon stocked walleye fry. Managers were interested in predicting how predation vulnerability may decrease with an increase in stocked walleye total length. We, therefore, use regressions developed here to estimate the prey total length distribution (e.g., Fig 4) for every smallmouth bass observed in the reservoir from 2011 to 2016 during routine UT-DWR sampling (n = 235). The sum of these distributions can be used to determine the size distribution of prey fishes likely to be consumed by the smallmouth bass population as a whole (Fig 7). Managers were informed that walleye stocked at 28 mm are likely to surpass ingested prey total lengths in 10% of encounters. Similarly, walleye stocked at 54, 92, and 131 mm are likely to surpass ingested prey total lengths in 50%, 90%, and 99% of encounters with smallmouth bass, respectively. UT-DWR managers and hatchery personnel are using these findings to inform stocking decisions.

## Conclusions and applications

Regressions derived in our study (Tables 2 and 3, S2 Appendix) can be used to predict, estimate, and model predator-prey total length relationships without the collection of additional data, saving researchers and managers an immeasurable amount of time, effort, and resources. As illustrated in the case study above, fisheries managers could use our models to optimize stocking efforts by minimizing the amount of time and money allocated toward rearing fish while maximizing the proportion of stocked fish likely to survive encounters with predators. This can be achieved using our models with very basic information about predator size structure (e.g., Fig 7). Similarly, estimating the potential impact of an invasive predator in a new ecosystem is another application of our models (e.g., [62]). Our models could also be applied in novel, individual-based modeling approaches for a variety of applications from understanding the role of rare, large diet items in fish growth and reproduction (e.g., [63]) to quantifying how changes in size structure of a predator population may release a prey population from density-dependent growth stunting. We have developed a web-based user interface to maximize the utility of our models that can be found at www.LakeEcologyLab.org/pred_prey. Users can download model predictions based on entered individual predator total lengths or upload a .csv file with lengths for an entire predator population. Ultimately, we hope managers and researchers use our models as tools to better understand, predict, and model predator-prey dynamics in aquatic ecosystems.

## Supporting information

S1 Appendix Body shape classification of prey taxa. Table A: Body shapes attributed to prey fish taxa used in our analyses.
(DOCX)

S2 Appendix 1^st^– 99^th^ percentile regression coefficients. Table A: Percentile regression coefficients for all-prey models. Table B: Quantile regression coefficients for prey-shape specific models.
(DOCX)

S3 Appendix Relative IP_max_ literature review. Table A: Review of gape-limit and maximum ingestible prey lengths estimates from the literature for our study piscivores. Fig A: Predator-specific maximum ingestible prey length.
(DOCX)

S4 Appendix Rock bass percentile regression evaluation. Fig A. Rock bass percentile regression evaluation.
(DOCX)
