# Supplementary material for: Go big or … don't? A field-based diet evaluation of freshwater piscivore and prey fish size relationships
Source: PLoS One. 2018 Mar 15;13(3):e0194092. doi: 10.1371/journal.pone.0194092 (PMC5854328; doi:10.1371/journal.pone.0194092)
Supplement: S1 Appendix — Table A: Body shapes attributed to prey fish taxa used in our analyses. (DOCX) [file pone.0194092.s001.docx]

**S1 Appendix. Body shape classification of prey taxa.**

**Table A**. **Body shapes attributed to prey fish taxa used in our analyses.**

| Taxanomic Group | Common Name | Body Shape |
| --- | --- | --- |
| Atherinidae | Silver sides | Fusiform |
| Catostomidae | Suckers | Fusiform |
| *Coregonus artedi* | Cisco | Fusiform |
| Cottidae | Sculpins | Fusiform |
| Cyprinidae | Minnows | Fusiform |
| Cyprinodontidae | Killifish | Fusiform |
| esocidae | Pikes | Fusiform |
| Fundulidae | Topminnows | Fusiform |
| Gasterosteidae | Sticklebacks | Fusiform |
| Ictaluridae | Catfishes | Fusiform |
| *Lota lota* | Burbot | Fusiform |
| *Micropterus* spp. | Black bass | Fusiform |
| *Osmerus mordax* | Rainbow smelt | Fusiform |
| Percidae | Perches | Fusiform |
| Percopsidae | Trout perches | Fusiform |
| Umbridae | Mudminnows | Fusiform |
| *Ambloplites rupestris* | Rock bass | Laterally compressed |
| *Dorosoma cepedianum* | Gizzard shad | Laterally compressed |
| *Lepomis* spp. | Sunfishes | Laterally compressed |
| Moronidae | Temperate bass | Laterally compressed |
| *Notemigonus crysoleucas* | Golden shiner | Laterally compressed |
| *Pomoxis* spp. | Crappies | Laterally compressed |
| Sciaenidae | Frshwater drum | Laterally compressed |
| Centrarchid | Sunfishes | Unknown |
| Unidentified | Unidentified | Unknown |
