# Supplementary material for: Go big or … don't? A field-based diet evaluation of freshwater piscivore and prey fish size relationships
Source: PLoS One. 2018 Mar 15;13(3):e0194092. doi: 10.1371/journal.pone.0194092 (PMC5854328; doi:10.1371/journal.pone.0194092)
Supplement: S2 Appendix — Table A: Percentile regression coefficients for all-prey models. Table B: Quantile regression coefficients for prey-shape specific models. (DOCX) [file pone.0194092.s002.docx]

**S2 Appendix. 1^st^ – 99^th^ percentile regression coefficients.**

**Table A. Percentile regression coefficients for all-prey models.**

Quantile regression coefficients for the 1^st^ – 99^th^ percentile regressions of prey total length (mm) as a function of predator total length (mm) for seven north temperate piscivores. Coefficients (*α* and *β*) correspond with equations in Table 2.

| Percentile | Crappie | | Largemouth Bass | | Muskellunge | | Northern Pike | | Rock Bass | | Smallmouth Bass | | Walleye | |
| --- | --- | --- | --- | --- | --- | --- | --- | --- | --- | --- | --- | --- | --- | --- |
|  | *α* | *β* | *α* | *β* | *α* | *β* | *α* | *β* | *α* | *β* | *α* | *β* | *α* | *β* |
| 1 | 0.96 | 1.07e-01 | 1.83 | 2.49e-03 | 2.99 | 1.17e-03 | -0.51 | 5.96e-01 | 3.09 | 6.78e-06 | 2.95 | 1.73e-06 | 2.54 | 1.29e-03 |
| 2 | 1.28 | 8.79e-02 | 1.92 | 2.59e-03 | 2.99 | 1.21e-03 | -1.10 | 6.99e-01 | 3.09 | 6.78e-06 | 3.00 | 1.68e-06 | 2.70 | 1.18e-03 |
| 3 | 1.31 | 8.99e-02 | 1.97 | 2.61e-03 | 2.94 | 1.37e-03 | 1.49 | 3.23e-01 | 3.15 | 5.67e-06 | 2.97 | 2.10e-06 | 2.79 | 1.17e-03 |
| 4 | 1.97 | 4.92e-02 | 1.81 | 3.64e-03 | 2.93 | 1.40e-03 | 2.11 | 2.39e-01 | 3.14 | 6.37e-06 | 2.99 | 2.26e-06 | 2.88 | 1.11e-03 |
| 5 | 1.95 | 5.14e-02 | 1.94 | 3.42e-03 | 2.90 | 1.46e-03 | 1.87 | 2.81e-01 | 3.14 | 6.37e-06 | 3.00 | 2.23e-06 | 2.92 | 1.10e-03 |
| 6 | 1.90 | 5.63e-02 | 1.93 | 3.66e-03 | 2.96 | 1.40e-03 | 1.57 | 3.35e-01 | 3.24 | 4.61e-06 | 3.05 | 2.21e-06 | 2.98 | 1.04e-03 |
| 7 | 1.90 | 5.63e-02 | 1.96 | 3.64e-03 | 2.99 | 1.35e-03 | 1.37 | 3.71e-01 | 3.24 | 4.61e-06 | 3.02 | 2.74e-06 | 3.02 | 1.04e-03 |
| 8 | 1.85 | 6.06e-02 | 1.98 | 3.64e-03 | 3.01 | 1.47e-03 | 1.13 | 4.15e-01 | 3.19 | 8.69e-06 | 3.03 | 2.82e-06 | 3.06 | 1.02e-03 |
| 9 | 1.87 | 5.92e-02 | 1.98 | 3.73e-03 | 3.05 | 1.43e-03 | 1.04 | 4.31e-01 | 3.19 | 8.97e-06 | 3.04 | 2.80e-06 | 3.08 | 1.03e-03 |
| 10 | 1.80 | 6.63e-02 | 1.95 | 4.04e-03 | 3.00 | 1.58e-03 | 0.95 | 4.48e-01 | 3.17 | 1.09e-05 | 3.07 | 2.71e-06 | 3.09 | 1.05e-03 |
| 11 | 1.77 | 6.89e-02 | 1.96 | 4.08e-03 | 3.01 | 1.61e-03 | 1.03 | 4.39e-01 | 3.14 | 1.23e-05 | 3.10 | 2.59e-06 | 3.12 | 1.02e-03 |
| 12 | 1.89 | 6.12e-02 | 2.02 | 4.01e-03 | 2.97 | 1.71e-03 | 0.83 | 4.73e-01 | 3.14 | 1.23e-05 | 3.13 | 2.70e-06 | 3.15 | 1.01e-03 |
| 13 | 1.94 | 5.88e-02 | 2.05 | 3.94e-03 | 2.98 | 1.74e-03 | 0.76 | 4.85e-01 | 3.15 | 1.19e-05 | 3.15 | 2.64e-06 | 3.16 | 1.03e-03 |
| 14 | 1.77 | 7.21e-02 | 2.08 | 3.90e-03 | 2.99 | 1.74e-03 | 1.51 | 3.74e-01 | 3.15 | 1.21e-05 | 3.15 | 2.78e-06 | 3.18 | 1.02e-03 |
| 15 | 1.83 | 6.87e-02 | 2.09 | 4.01e-03 | 3.02 | 1.73e-03 | 1.55 | 3.70e-01 | 3.15 | 1.21e-05 | 3.16 | 2.91e-06 | 3.21 | 9.89e-04 |
| 16 | 1.85 | 6.78e-02 | 2.13 | 3.94e-03 | 3.04 | 1.73e-03 | 1.47 | 3.86e-01 | 3.16 | 1.20e-05 | 3.18 | 2.90e-06 | 3.23 | 9.92e-04 |
| 17 | 1.73 | 7.70e-02 | 2.15 | 3.90e-03 | 3.03 | 1.76e-03 | 1.74 | 3.46e-01 | 3.16 | 1.20e-05 | 3.17 | 3.06e-06 | 3.23 | 1.02e-03 |
| 18 | 1.67 | 8.16e-02 | 2.17 | 3.89e-03 | 3.01 | 1.84e-03 | 1.99 | 3.09e-01 | 3.13 | 1.45e-05 | 3.27 | 2.67e-06 | 3.26 | 9.89e-04 |
| 19 | 1.67 | 8.14e-02 | 2.21 | 3.81e-03 | 2.99 | 1.89e-03 | 1.93 | 3.20e-01 | 3.12 | 1.47e-05 | 3.32 | 2.41e-06 | 3.29 | 9.59e-04 |
| 20 | 1.66 | 8.25e-02 | 2.22 | 3.86e-03 | 2.97 | 1.99e-03 | 1.91 | 3.24e-01 | 3.13 | 1.46e-05 | 3.41 | 2.02e-06 | 3.29 | 9.81e-04 |
| 21 | 1.58 | 8.93e-02 | 2.25 | 3.84e-03 | 2.97 | 2.04e-03 | 1.83 | 3.38e-01 | 3.13 | 1.46e-05 | 3.47 | 2.01e-06 | 3.30 | 9.87e-04 |
| 22 | 1.47 | 9.85e-02 | 2.26 | 3.87e-03 | 2.95 | 2.09e-03 | 1.78 | 3.48e-01 | 3.13 | 1.46e-05 | 3.49 | 2.00e-06 | 3.31 | 9.83e-04 |
| 23 | 1.56 | 9.25e-02 | 2.27 | 3.94e-03 | 2.96 | 2.10e-03 | 1.72 | 3.59e-01 | 3.13 | 1.47e-05 | 3.51 | 1.92e-06 | 3.33 | 9.66e-04 |
| 24 | 1.51 | 9.71e-02 | 2.29 | 3.90e-03 | 2.96 | 2.12e-03 | 1.79 | 3.50e-01 | 3.13 | 1.47e-05 | 3.52 | 2.09e-06 | 3.33 | 9.80e-04 |
| 25 | 1.40 | 1.07e-01 | 2.32 | 3.85e-03 | 3.00 | 2.09e-03 | 1.72 | 3.62e-01 | 3.13 | 1.52e-05 | 3.57 | 1.83e-06 | 3.33 | 1.00e-03 |
| 26 | 1.26 | 1.18e-01 | 2.32 | 3.93e-03 | 3.01 | 2.09e-03 | 1.68 | 3.70e-01 | 3.13 | 1.52e-05 | 3.58 | 1.87e-06 | 3.34 | 1.02e-03 |
| 27 | 1.22 | 1.22e-01 | 2.35 | 3.89e-03 | 2.99 | 2.15e-03 | 1.64 | 3.78e-01 | 3.19 | 1.40e-05 | 3.61 | 1.82e-06 | 3.35 | 1.00e-03 |
| 28 | 1.17 | 1.27e-01 | 2.35 | 3.94e-03 | 2.99 | 2.18e-03 | 1.55 | 3.92e-01 | 3.19 | 1.40e-05 | 3.63 | 1.72e-06 | 3.36 | 9.93e-04 |
| 29 | 1.13 | 1.30e-01 | 2.37 | 3.98e-03 | 2.99 | 2.22e-03 | 1.51 | 4.01e-01 | 3.17 | 1.54e-05 | 3.67 | 1.62e-06 | 3.38 | 9.75e-04 |
| 30 | 1.10 | 1.34e-01 | 2.38 | 3.97e-03 | 2.94 | 2.35e-03 | 1.55 | 3.95e-01 | 3.17 | 1.54e-05 | 3.68 | 1.67e-06 | 3.40 | 9.60e-04 |
| 31 | 1.06 | 1.37e-01 | 2.40 | 3.99e-03 | 2.97 | 2.32e-03 | 1.48 | 4.07e-01 | 3.21 | 1.44e-05 | 3.71 | 1.53e-06 | 3.41 | 9.57e-04 |
| 32 | 1.03 | 1.40e-01 | 2.43 | 3.93e-03 | 2.96 | 2.35e-03 | 1.46 | 4.12e-01 | 3.21 | 1.44e-05 | 3.72 | 1.56e-06 | 3.42 | 9.40e-04 |
| 33 | 1.06 | 1.38e-01 | 2.43 | 3.99e-03 | 2.99 | 2.33e-03 | 1.52 | 4.03e-01 | 3.22 | 1.43e-05 | 3.72 | 1.55e-06 | 3.43 | 9.42e-04 |
| 34 | 1.10 | 1.37e-01 | 2.44 | 3.98e-03 | 2.99 | 2.34e-03 | 1.48 | 4.10e-01 | 3.22 | 1.44e-05 | 3.72 | 1.58e-06 | 3.44 | 9.44e-04 |
| 35 | 1.08 | 1.39e-01 | 2.45 | 4.02e-03 | 2.97 | 2.40e-03 | 1.52 | 4.05e-01 | 3.23 | 1.42e-05 | 3.72 | 1.61e-06 | 3.45 | 9.38e-04 |
| 36 | 1.05 | 1.43e-01 | 2.46 | 3.99e-03 | 3.03 | 2.34e-03 | 1.61 | 3.93e-01 | 3.23 | 1.42e-05 | 3.75 | 1.44e-06 | 3.47 | 9.35e-04 |
| 37 | 1.05 | 1.43e-01 | 2.47 | 4.00e-03 | 3.04 | 2.35e-03 | 1.67 | 3.83e-01 | 3.23 | 1.42e-05 | 3.76 | 1.42e-06 | 3.48 | 9.29e-04 |
| 38 | 1.00 | 1.46e-01 | 2.48 | 4.05e-03 | 3.06 | 2.35e-03 | 2.02 | 3.33e-01 | 3.22 | 1.54e-05 | 3.76 | 1.55e-06 | 3.48 | 9.38e-04 |
| 39 | 0.92 | 1.53e-01 | 2.50 | 4.02e-03 | 3.11 | 2.29e-03 | 1.97 | 3.42e-01 | 3.22 | 1.54e-05 | 3.76 | 1.64e-06 | 3.49 | 9.31e-04 |
| 40 | 1.06 | 1.45e-01 | 2.51 | 4.03e-03 | 3.11 | 2.29e-03 | 1.94 | 3.48e-01 | 3.22 | 1.54e-05 | 3.76 | 1.65e-06 | 3.50 | 9.22e-04 |
| 41 | 1.15 | 1.40e-01 | 2.52 | 4.03e-03 | 3.11 | 2.33e-03 | 1.92 | 3.52e-01 | 3.21 | 1.62e-05 | 3.77 | 1.65e-06 | 3.51 | 9.31e-04 |
| 42 | 1.17 | 1.39e-01 | 2.53 | 4.02e-03 | 3.14 | 2.31e-03 | 1.91 | 3.55e-01 | 3.21 | 1.62e-05 | 3.77 | 1.77e-06 | 3.51 | 9.37e-04 |
| 43 | 1.23 | 1.35e-01 | 2.55 | 4.01e-03 | 3.17 | 2.28e-03 | 1.88 | 3.60e-01 | 3.23 | 1.59e-05 | 3.77 | 1.80e-06 | 3.52 | 9.44e-04 |
| 44 | 1.31 | 1.31e-01 | 2.55 | 4.05e-03 | 3.18 | 2.29e-03 | 1.83 | 3.70e-01 | 3.23 | 1.59e-05 | 3.78 | 1.79e-06 | 3.53 | 9.24e-04 |
| 45 | 1.27 | 1.36e-01 | 2.60 | 3.96e-03 | 3.17 | 2.33e-03 | 1.76 | 3.82e-01 | 3.23 | 1.58e-05 | 3.79 | 1.77e-06 | 3.55 | 9.05e-04 |
| 46 | 1.26 | 1.37e-01 | 2.61 | 3.94e-03 | 3.20 | 2.29e-03 | 1.79 | 3.79e-01 | 3.21 | 1.68e-05 | 3.82 | 1.62e-06 | 3.55 | 9.07e-04 |
| 47 | 1.05 | 1.53e-01 | 2.61 | 4.00e-03 | 3.20 | 2.30e-03 | 1.74 | 3.89e-01 | 3.22 | 1.67e-05 | 3.81 | 1.73e-06 | 3.56 | 8.99e-04 |
| 48 | 1.07 | 1.54e-01 | 2.61 | 4.01e-03 | 3.23 | 2.27e-03 | 1.66 | 4.04e-01 | 3.22 | 1.67e-05 | 3.82 | 1.71e-06 | 3.57 | 9.06e-04 |
| 49 | 1.08 | 1.54e-01 | 2.63 | 3.97e-03 | 3.23 | 2.29e-03 | 1.69 | 4.01e-01 | 3.23 | 1.64e-05 | 3.82 | 1.78e-06 | 3.58 | 9.17e-04 |
| 50 | 1.07 | 1.55e-01 | 2.65 | 3.94e-03 | 3.23 | 2.31e-03 | 1.62 | 4.13e-01 | 3.23 | 1.64e-05 | 3.83 | 1.82e-06 | 3.59 | 9.06e-04 |
| 51 | 1.03 | 1.59e-01 | 2.66 | 3.99e-03 | 3.24 | 2.31e-03 | 1.63 | 4.16e-01 | 3.23 | 1.68e-05 | 3.83 | 2.01e-06 | 3.60 | 9.13e-04 |
| 52 | 1.02 | 1.61e-01 | 2.68 | 3.94e-03 | 3.25 | 2.31e-03 | 1.56 | 4.28e-01 | 3.22 | 1.72e-05 | 3.85 | 1.90e-06 | 3.60 | 9.27e-04 |
| 53 | 1.23 | 1.49e-01 | 2.68 | 4.00e-03 | 3.27 | 2.28e-03 | 1.45 | 4.47e-01 | 3.22 | 1.72e-05 | 3.85 | 1.91e-06 | 3.60 | 9.41e-04 |
| 54 | 1.08 | 1.61e-01 | 2.69 | 4.00e-03 | 3.33 | 2.23e-03 | 1.38 | 4.59e-01 | 3.21 | 1.86e-05 | 3.85 | 2.06e-06 | 3.61 | 9.48e-04 |
| 55 | 1.06 | 1.63e-01 | 2.71 | 3.96e-03 | 3.34 | 2.21e-03 | 1.42 | 4.56e-01 | 3.24 | 1.77e-05 | 3.86 | 2.05e-06 | 3.62 | 9.58e-04 |
| 56 | 1.10 | 1.61e-01 | 2.70 | 4.05e-03 | 3.37 | 2.19e-03 | 1.31 | 4.76e-01 | 3.24 | 1.83e-05 | 3.88 | 2.04e-06 | 3.62 | 9.78e-04 |
| 57 | 1.18 | 1.57e-01 | 2.71 | 4.09e-03 | 3.46 | 2.08e-03 | 1.20 | 4.96e-01 | 3.24 | 1.83e-05 | 3.90 | 1.96e-06 | 3.62 | 1.00e-03 |
| 58 | 1.15 | 1.60e-01 | 2.71 | 4.13e-03 | 3.52 | 2.03e-03 | 1.18 | 5.02e-01 | 3.25 | 1.81e-05 | 3.91 | 2.00e-06 | 3.62 | 1.03e-03 |
| 59 | 1.15 | 1.60e-01 | 2.71 | 4.17e-03 | 3.54 | 2.01e-03 | 1.06 | 5.23e-01 | 3.25 | 1.81e-05 | 3.91 | 1.98e-06 | 3.62 | 1.04e-03 |
| 60 | 1.19 | 1.58e-01 | 2.70 | 4.26e-03 | 3.56 | 2.00e-03 | 0.93 | 5.46e-01 | 3.27 | 1.78e-05 | 3.93 | 1.94e-06 | 3.63 | 1.06e-03 |
| 61 | 1.27 | 1.55e-01 | 2.71 | 4.32e-03 | 3.58 | 1.99e-03 | 0.99 | 5.40e-01 | 3.27 | 1.80e-05 | 3.93 | 1.92e-06 | 3.63 | 1.09e-03 |
| 62 | 1.27 | 1.56e-01 | 2.74 | 4.27e-03 | 3.59 | 2.00e-03 | 0.86 | 5.62e-01 | 3.30 | 1.74e-05 | 3.94 | 1.92e-06 | 3.63 | 1.12e-03 |
| 63 | 1.36 | 1.51e-01 | 2.75 | 4.26e-03 | 3.59 | 2.01e-03 | 0.67 | 5.98e-01 | 3.30 | 1.74e-05 | 3.94 | 1.92e-06 | 3.63 | 1.15e-03 |
| 64 | 1.34 | 1.52e-01 | 2.77 | 4.25e-03 | 3.62 | 1.98e-03 | 0.49 | 6.30e-01 | 3.30 | 1.73e-05 | 3.95 | 1.93e-06 | 3.63 | 1.18e-03 |
| 65 | 1.53 | 1.41e-01 | 2.79 | 4.22e-03 | 3.60 | 2.05e-03 | 0.42 | 6.43e-01 | 3.30 | 1.73e-05 | 3.96 | 1.87e-06 | 3.64 | 1.17e-03 |
| 66 | 1.43 | 1.50e-01 | 2.80 | 4.21e-03 | 3.61 | 2.06e-03 | 0.34 | 6.58e-01 | 3.30 | 1.78e-05 | 3.97 | 1.89e-06 | 3.66 | 1.15e-03 |
| 67 | 1.48 | 1.47e-01 | 2.80 | 4.26e-03 | 3.63 | 2.05e-03 | 0.13 | 6.95e-01 | 3.30 | 1.78e-05 | 3.98 | 1.85e-06 | 3.67 | 1.14e-03 |
| 68 | 1.45 | 1.49e-01 | 2.82 | 4.26e-03 | 3.66 | 2.01e-03 | 0.01 | 7.16e-01 | 3.30 | 1.78e-05 | 4.00 | 1.79e-06 | 3.68 | 1.12e-03 |
| 69 | 1.50 | 1.47e-01 | 2.84 | 4.22e-03 | 3.67 | 2.01e-03 | -0.09 | 7.33e-01 | 3.30 | 1.78e-05 | 4.00 | 1.90e-06 | 3.70 | 1.10e-03 |
| 70 | 1.52 | 1.46e-01 | 2.88 | 4.17e-03 | 3.68 | 1.99e-03 | -0.32 | 7.73e-01 | 3.34 | 1.71e-05 | 3.99 | 2.02e-06 | 3.71 | 1.08e-03 |
| 71 | 1.52 | 1.47e-01 | 2.91 | 4.13e-03 | 3.73 | 1.95e-03 | -0.58 | 8.20e-01 | 3.34 | 1.71e-05 | 3.99 | 2.08e-06 | 3.73 | 1.06e-03 |
| 72 | 1.57 | 1.45e-01 | 2.92 | 4.10e-03 | 3.73 | 1.96e-03 | -0.68 | 8.38e-01 | 3.35 | 1.75e-05 | 4.01 | 1.98e-06 | 3.74 | 1.04e-03 |
| 73 | 1.61 | 1.44e-01 | 2.93 | 4.15e-03 | 3.76 | 1.94e-03 | -0.86 | 8.70e-01 | 3.35 | 1.75e-05 | 4.03 | 1.91e-06 | 3.76 | 1.02e-03 |
| 74 | 1.59 | 1.46e-01 | 2.94 | 4.18e-03 | 3.79 | 1.93e-03 | -1.08 | 9.10e-01 | 3.35 | 1.75e-05 | 4.05 | 1.91e-06 | 3.77 | 1.01e-03 |
| 75 | 1.66 | 1.42e-01 | 2.95 | 4.20e-03 | 3.80 | 1.95e-03 | -1.29 | 9.49e-01 | 3.42 | 1.62e-05 | 4.05 | 1.98e-06 | 3.79 | 9.82e-04 |
| 76 | 1.61 | 1.47e-01 | 2.96 | 4.20e-03 | 3.88 | 1.86e-03 | -1.62 | 1.01e+00 | 3.39 | 1.88e-05 | 4.06 | 1.93e-06 | 3.80 | 9.67e-04 |
| 77 | 1.66 | 1.44e-01 | 2.99 | 4.15e-03 | 3.92 | 1.81e-03 | -1.79 | 1.04e+00 | 3.39 | 1.91e-05 | 4.06 | 2.05e-06 | 3.81 | 9.60e-04 |
| 78 | 1.81 | 1.35e-01 | 2.99 | 4.23e-03 | 3.91 | 1.83e-03 | -2.01 | 1.08e+00 | 3.39 | 1.91e-05 | 4.06 | 2.15e-06 | 3.82 | 9.52e-04 |
| 79 | 1.75 | 1.40e-01 | 3.02 | 4.16e-03 | 3.88 | 1.94e-03 | -2.19 | 1.11e+00 | 3.39 | 1.91e-05 | 4.09 | 2.09e-06 | 3.85 | 9.24e-04 |
| 80 | 1.76 | 1.41e-01 | 3.05 | 4.12e-03 | 3.95 | 1.85e-03 | -2.32 | 1.14e+00 | 3.39 | 1.91e-05 | 4.13 | 1.99e-06 | 3.84 | 9.78e-04 |
| 81 | 1.85 | 1.36e-01 | 3.07 | 4.10e-03 | 3.99 | 1.81e-03 | -2.48 | 1.16e+00 | 3.46 | 1.79e-05 | 4.13 | 1.97e-06 | 3.84 | 1.03e-03 |
| 82 | 1.94 | 1.31e-01 | 3.07 | 4.16e-03 | 4.02 | 1.79e-03 | -2.75 | 1.21e+00 | 3.51 | 1.71e-05 | 4.15 | 1.92e-06 | 3.85 | 1.04e-03 |
| 83 | 1.94 | 1.32e-01 | 3.08 | 4.17e-03 | 4.03 | 1.78e-03 | -3.00 | 1.26e+00 | 3.50 | 1.78e-05 | 4.17 | 1.84e-06 | 3.87 | 1.01e-03 |
| 84 | 1.92 | 1.34e-01 | 3.09 | 4.20e-03 | 4.06 | 1.78e-03 | -3.00 | 1.26e+00 | 3.50 | 1.78e-05 | 4.21 | 1.67e-06 | 3.87 | 1.07e-03 |
| 85 | 1.92 | 1.36e-01 | 3.11 | 4.21e-03 | 4.08 | 1.78e-03 | -3.00 | 1.27e+00 | 3.49 | 1.90e-05 | 4.22 | 1.71e-06 | 3.87 | 1.12e-03 |
| 86 | 2.01 | 1.30e-01 | 3.11 | 4.24e-03 | 4.04 | 1.91e-03 | -3.26 | 1.31e+00 | 3.47 | 2.15e-05 | 4.25 | 1.85e-06 | 3.89 | 1.09e-03 |
| 87 | 2.04 | 1.29e-01 | 3.12 | 4.25e-03 | 4.03 | 1.95e-03 | -3.49 | 1.36e+00 | 3.47 | 2.15e-05 | 4.30 | 1.77e-06 | 3.91 | 1.09e-03 |
| 88 | 1.91 | 1.39e-01 | 3.15 | 4.20e-03 | 4.04 | 1.95e-03 | -3.40 | 1.35e+00 | 3.48 | 2.13e-05 | 4.32 | 1.70e-06 | 3.92 | 1.14e-03 |
| 89 | 2.22 | 1.21e-01 | 3.19 | 4.15e-03 | 4.07 | 1.94e-03 | -3.47 | 1.36e+00 | 3.48 | 2.13e-05 | 4.36 | 1.60e-06 | 3.92 | 1.21e-03 |
| 90 | 2.20 | 1.22e-01 | 3.19 | 4.18e-03 | 4.12 | 1.90e-03 | -3.58 | 1.38e+00 | 3.52 | 2.05e-05 | 4.36 | 1.68e-06 | 3.92 | 1.29e-03 |
| 91 | 2.16 | 1.26e-01 | 3.26 | 4.00e-03 | 4.15 | 1.89e-03 | -3.64 | 1.39e+00 | 3.66 | 1.80e-05 | 4.37 | 1.94e-06 | 3.93 | 1.33e-03 |
| 92 | 2.13 | 1.30e-01 | 3.33 | 3.85e-03 | 4.14 | 1.92e-03 | -3.67 | 1.40e+00 | 3.66 | 1.80e-05 | 4.40 | 2.07e-06 | 3.94 | 1.39e-03 |
| 93 | 2.02 | 1.39e-01 | 3.36 | 3.81e-03 | 4.17 | 1.91e-03 | -3.76 | 1.42e+00 | 3.81 | 1.59e-05 | 4.48 | 1.89e-06 | 3.94 | 1.49e-03 |
| 94 | 2.04 | 1.39e-01 | 3.38 | 3.83e-03 | 4.23 | 1.85e-03 | -3.84 | 1.43e+00 | 3.81 | 1.59e-05 | 4.53 | 1.74e-06 | 3.94 | 1.60e-03 |
| 95 | 1.93 | 1.48e-01 | 3.41 | 1.21e-01 | 4.28 | 1.87e-03 | 4.63 | 1.44e+00 | 3.84 | 1.55e-05 | 4.55 | 1.72e-06 | 3.94 | 1.73e-03 |
| 96 | 2.07 | 1.40e-01 | 3.46 | 1.17e-01 | 4.33 | 1.83e-03 | 4.67 | 1.47e+00 | 3.84 | 1.55e-05 | 4.61 | 1.45e-06 | 3.98 | 1.72e-03 |
| 97 | 2.03 | 1.43e-01 | 3.53 | 1.19e-01 | 4.36 | 1.79e-03 | 4.76 | 1.47e+00 | 3.82 | 1.70e-05 | 4.54 | 2.58e-06 | 4.02 | 1.75e-03 |
| 98 | 2.41 | 1.24e-01 | 3.60 | 1.10e-01 | 4.53 | 1.61e-03 | 4.82 | 1.51e+00 | 4.13 | 1.13e-05 | 4.57 | 2.71e-06 | 4.06 | 1.81e-03 |
| 99 | 1.85 | 1.69e-01 | 3.61 | 1.11e-01 | 4.48 | 1.76e-03 | 4.94 | 1.43e+00 | 4.13 | 1.13e-05 | 4.64 | 2.48e-06 | 4.07 | 2.00e-03 |

**Table B. Quantile regression coefficients for prey-shape specific models.**

Quantile regression coefficients for the 1^st^ – 99^th^ percentile regressions of prey total length (mm) as a function of predator total length (mm) for fusiform and laterally compressed prey of three north temperate piscivores. Coefficients (*α* and *β*) correspond with equations in Table 2.

| Percentile | Largemouth Bass | | | | Muskellunge | | | | Walleye | | | |
| --- | --- | --- | --- | --- | --- | --- | --- | --- | --- | --- | --- | --- |
|  | Fusiform | | Laterally Compressed | | Fusiform | | Laterally Compressed | | Fusiform | | Laterally Compressed | |
|  | *α* | *β* | *α* | *β* | *α* | *β* | *α* | *β* | *α* | *β* | *α* | *β* |
| 1 | -0.33 | 5.67e-01 | 2.00 | 7.47e-06 | 2.95 | 1.15e-03 | 1.13 | 9.26e-03 | 2.69 | 1.37e-03 | 2.21 | 3.52e-06 |
| 2 | 1.88 | 2.40e-01 | 2.19 | 6.25e-06 | 3.10 | 9.91e-04 | 1.13 | 9.26e-03 | 2.70 | 1.49e-03 | 2.36 | 3.24e-06 |
| 3 | 2.28 | 1.79e-01 | 2.16 | 8.54e-06 | 3.09 | 1.10e-03 | 2.96 | 6.64e-03 | 2.85 | 1.33e-03 | 2.40 | 3.17e-06 |
| 4 | 2.29 | 1.84e-01 | 2.20 | 8.43e-06 | 3.15 | 1.02e-03 | 2.68 | 7.33e-03 | 2.90 | 1.32e-03 | 2.53 | 2.94e-06 |
| 5 | 2.04 | 2.38e-01 | 2.16 | 1.04e-05 | 3.25 | 9.22e-04 | 2.68 | 7.33e-03 | 2.93 | 1.31e-03 | 2.56 | 2.89e-06 |
| 6 | 1.76 | 2.98e-01 | 2.18 | 1.08e-05 | 3.25 | 9.22e-04 | 1.62 | 1.02e-02 | 2.99 | 1.24e-03 | 2.61 | 2.80e-06 |
| 7 | 1.67 | 3.17e-01 | 2.23 | 1.07e-05 | 3.25 | 9.22e-04 | 2.18 | 9.50e-03 | 3.00 | 1.28e-03 | 2.64 | 2.74e-06 |
| 8 | 1.62 | 3.28e-01 | 2.24 | 1.06e-05 | 3.27 | 9.55e-04 | 2.18 | 9.50e-03 | 3.03 | 1.27e-03 | 2.68 | 2.67e-06 |
| 9 | 1.29 | 3.99e-01 | 2.25 | 1.10e-05 | 3.27 | 9.53e-04 | 2.23 | 9.56e-03 | 3.08 | 1.21e-03 | 2.70 | 2.64e-06 |
| 10 | 1.26 | 4.07e-01 | 2.27 | 1.09e-05 | 3.40 | 8.16e-04 | 2.23 | 9.56e-03 | 3.11 | 1.19e-03 | 2.74 | 2.57e-06 |
| 11 | 1.26 | 4.11e-01 | 2.28 | 1.11e-05 | 3.41 | 8.25e-04 | 2.42 | 9.34e-03 | 3.14 | 1.16e-03 | 2.75 | 2.55e-06 |
| 12 | 1.26 | 4.16e-01 | 2.32 | 1.09e-05 | 3.34 | 1.04e-03 | 2.42 | 9.34e-03 | 3.16 | 1.15e-03 | 2.80 | 2.46e-06 |
| 13 | 1.21 | 4.26e-01 | 2.34 | 1.11e-05 | 3.30 | 1.14e-03 | 2.41 | 9.45e-03 | 3.20 | 1.10e-03 | 2.84 | 2.40e-06 |
| 14 | 1.14 | 4.42e-01 | 2.35 | 1.15e-05 | 3.24 | 1.30e-03 | 2.41 | 9.45e-03 | 3.22 | 1.09e-03 | 2.88 | 2.32e-06 |
| 15 | 1.12 | 4.47e-01 | 2.37 | 1.13e-05 | 3.20 | 1.41e-03 | 2.69 | 9.11e-03 | 3.24 | 1.08e-03 | 2.88 | 2.32e-06 |
| 16 | 1.27 | 4.21e-01 | 2.37 | 1.14e-05 | 3.15 | 1.56e-03 | 2.59 | 9.43e-03 | 3.27 | 1.05e-03 | 2.88 | 2.32e-06 |
| 17 | 1.65 | 3.60e-01 | 2.37 | 1.16e-05 | 3.13 | 1.59e-03 | 2.80 | 9.18e-03 | 3.28 | 1.04e-03 | 2.90 | 2.29e-06 |
| 18 | 1.79 | 3.39e-01 | 2.38 | 1.16e-05 | 3.12 | 1.63e-03 | 2.67 | 9.55e-03 | 3.30 | 1.04e-03 | 3.00 | 2.11e-06 |
| 19 | 1.78 | 3.41e-01 | 2.40 | 1.15e-05 | 3.04 | 1.86e-03 | 2.67 | 9.55e-03 | 3.32 | 1.01e-03 | 3.02 | 2.07e-06 |
| 20 | 1.75 | 3.49e-01 | 2.43 | 1.14e-05 | 2.92 | 2.19e-03 | 2.65 | 9.62e-03 | 3.33 | 1.01e-03 | 3.09 | 1.95e-06 |
| 21 | 1.74 | 3.50e-01 | 2.45 | 1.13e-05 | 2.91 | 2.22e-03 | 2.78 | 9.47e-03 | 3.33 | 1.02e-03 | 3.09 | 1.95e-06 |
| 22 | 1.74 | 3.51e-01 | 2.47 | 1.12e-05 | 2.84 | 2.41e-03 | 2.78 | 9.47e-03 | 3.34 | 1.01e-03 | 3.17 | 1.81e-06 |
| 23 | 1.72 | 3.54e-01 | 2.49 | 1.11e-05 | 2.80 | 2.51e-03 | 2.78 | 9.47e-03 | 3.35 | 1.01e-03 | 3.18 | 1.80e-06 |
| 24 | 1.70 | 3.59e-01 | 2.50 | 1.10e-05 | 2.88 | 2.44e-03 | 2.75 | 9.61e-03 | 3.36 | 9.98e-04 | 3.20 | 1.75e-06 |
| 25 | 1.66 | 3.70e-01 | 2.54 | 1.08e-05 | 2.88 | 2.43e-03 | 3.16 | 9.17e-03 | 3.38 | 9.84e-04 | 3.20 | 1.75e-06 |
| 26 | 1.62 | 3.79e-01 | 2.55 | 1.08e-05 | 2.92 | 2.39e-03 | 3.16 | 9.17e-03 | 3.39 | 9.84e-04 | 3.20 | 1.75e-06 |
| 27 | 1.61 | 3.82e-01 | 2.57 | 1.07e-05 | 2.96 | 2.36e-03 | 3.19 | 9.14e-03 | 3.40 | 9.77e-04 | 3.20 | 1.75e-06 |
| 28 | 1.58 | 3.88e-01 | 2.58 | 1.06e-05 | 2.96 | 2.35e-03 | 3.19 | 9.14e-03 | 3.42 | 9.58e-04 | 3.20 | 1.75e-06 |
| 29 | 1.56 | 3.92e-01 | 2.59 | 1.06e-05 | 3.00 | 2.31e-03 | 3.28 | 9.05e-03 | 3.43 | 9.53e-04 | 3.20 | 1.75e-06 |
| 30 | 1.61 | 3.85e-01 | 2.59 | 1.06e-05 | 3.05 | 2.27e-03 | 3.28 | 9.05e-03 | 3.45 | 9.33e-04 | 3.20 | 1.75e-06 |
| 31 | 1.57 | 3.95e-01 | 2.61 | 1.05e-05 | 3.05 | 2.27e-03 | 3.35 | 8.98e-03 | 3.48 | 9.05e-04 | 3.20 | 1.75e-06 |
| 32 | 1.55 | 4.00e-01 | 2.61 | 1.05e-05 | 3.11 | 2.21e-03 | 3.35 | 8.98e-03 | 3.48 | 9.19e-04 | 3.20 | 1.75e-06 |
| 33 | 1.52 | 4.06e-01 | 2.62 | 1.04e-05 | 3.16 | 2.16e-03 | 3.35 | 8.98e-03 | 3.49 | 9.25e-04 | 3.20 | 1.79e-06 |
| 34 | 1.51 | 4.08e-01 | 2.64 | 1.03e-05 | 3.21 | 2.11e-03 | 3.39 | 9.17e-03 | 3.49 | 9.35e-04 | 3.20 | 2.28e-06 |
| 35 | 1.49 | 4.13e-01 | 2.66 | 1.03e-05 | 3.24 | 2.08e-03 | 3.39 | 9.17e-03 | 3.51 | 9.17e-04 | 3.19 | 2.85e-06 |
| 36 | 1.46 | 4.19e-01 | 2.67 | 1.02e-05 | 3.26 | 2.06e-03 | 3.52 | 9.11e-03 | 3.51 | 9.36e-04 | 3.19 | 2.87e-06 |
| 37 | 1.44 | 4.24e-01 | 2.68 | 1.01e-05 | 3.28 | 2.03e-03 | 3.25 | 9.86e-03 | 3.51 | 9.41e-04 | 3.19 | 2.94e-06 |
| 38 | 1.41 | 4.29e-01 | 2.68 | 1.05e-05 | 3.33 | 1.99e-03 | 3.32 | 9.79e-03 | 3.52 | 9.43e-04 | 3.19 | 2.94e-06 |
| 39 | 1.40 | 4.35e-01 | 2.68 | 1.08e-05 | 3.31 | 2.06e-03 | 3.32 | 9.79e-03 | 3.53 | 9.24e-04 | 3.19 | 3.10e-06 |
| 40 | 1.39 | 4.37e-01 | 2.68 | 1.10e-05 | 3.34 | 2.03e-03 | 3.29 | 9.87e-03 | 3.55 | 9.04e-04 | 3.19 | 3.28e-06 |
| 41 | 1.41 | 4.34e-01 | 2.71 | 1.09e-05 | 3.34 | 2.06e-03 | 3.42 | 9.70e-03 | 3.55 | 9.05e-04 | 3.18 | 3.55e-06 |
| 42 | 1.50 | 4.21e-01 | 2.72 | 1.09e-05 | 3.33 | 2.08e-03 | 2.99 | 1.09e-02 | 3.56 | 9.02e-04 | 3.18 | 3.55e-06 |
| 43 | 1.49 | 4.23e-01 | 2.74 | 1.08e-05 | 3.35 | 2.08e-03 | 3.32 | 1.05e-02 | 3.57 | 8.98e-04 | 3.18 | 3.70e-06 |
| 44 | 1.57 | 4.11e-01 | 2.75 | 1.08e-05 | 3.36 | 2.08e-03 | 3.32 | 1.05e-02 | 3.58 | 8.99e-04 | 3.18 | 3.70e-06 |
| 45 | 1.58 | 4.13e-01 | 2.76 | 1.07e-05 | 3.36 | 2.11e-03 | 3.64 | 1.01e-02 | 3.59 | 8.96e-04 | 3.18 | 3.70e-06 |
| 46 | 1.56 | 4.17e-01 | 2.77 | 1.07e-05 | 3.38 | 2.10e-03 | 3.57 | 1.03e-02 | 3.60 | 8.96e-04 | 3.18 | 3.70e-06 |
| 47 | 1.61 | 4.12e-01 | 2.80 | 1.06e-05 | 3.39 | 2.11e-03 | 3.57 | 1.03e-02 | 3.60 | 9.06e-04 | 3.18 | 3.70e-06 |
| 48 | 1.63 | 4.10e-01 | 2.81 | 1.05e-05 | 3.48 | 2.02e-03 | 3.59 | 1.04e-02 | 3.60 | 9.19e-04 | 3.18 | 3.73e-06 |
| 49 | 1.61 | 4.14e-01 | 2.82 | 1.05e-05 | 3.52 | 1.97e-03 | 3.59 | 1.04e-02 | 3.61 | 9.25e-04 | 3.18 | 3.73e-06 |
| 50 | 1.56 | 4.25e-01 | 2.83 | 1.04e-05 | 3.52 | 1.98e-03 | 3.57 | 1.05e-02 | 3.62 | 9.24e-04 | 3.18 | 3.73e-06 |
| 51 | 1.64 | 4.13e-01 | 2.86 | 1.03e-05 | 3.55 | 1.95e-03 | 3.56 | 1.05e-02 | 3.62 | 9.45e-04 | 3.21 | 3.66e-06 |
| 52 | 1.64 | 4.15e-01 | 2.88 | 1.02e-05 | 3.56 | 1.94e-03 | 3.56 | 1.05e-02 | 3.62 | 9.62e-04 | 3.23 | 3.60e-06 |
| 53 | 1.60 | 4.23e-01 | 2.88 | 1.02e-05 | 3.55 | 1.98e-03 | 3.78 | 1.03e-02 | 3.63 | 9.70e-04 | 3.26 | 3.55e-06 |
| 54 | 1.54 | 4.35e-01 | 2.89 | 1.01e-05 | 3.58 | 1.95e-03 | 3.78 | 1.03e-02 | 3.63 | 9.83e-04 | 3.29 | 3.46e-06 |
| 55 | 1.46 | 4.52e-01 | 2.90 | 1.01e-05 | 3.61 | 1.92e-03 | 3.78 | 1.03e-02 | 3.64 | 9.97e-04 | 3.31 | 3.42e-06 |
| 56 | 1.40 | 4.64e-01 | 2.91 | 1.00e-05 | 3.61 | 1.94e-03 | 3.71 | 1.05e-02 | 3.64 | 1.02e-03 | 3.31 | 3.42e-06 |
| 57 | 1.36 | 4.74e-01 | 2.94 | 9.91e-06 | 3.60 | 1.96e-03 | 3.93 | 1.02e-02 | 3.64 | 1.04e-03 | 3.33 | 3.38e-06 |
| 58 | 1.20 | 5.06e-01 | 2.97 | 9.78e-06 | 3.59 | 2.00e-03 | 3.79 | 1.06e-02 | 3.65 | 1.06e-03 | 3.40 | 3.22e-06 |
| 59 | 1.16 | 5.14e-01 | 2.98 | 9.75e-06 | 3.57 | 2.08e-03 | 3.79 | 1.06e-02 | 3.64 | 1.09e-03 | 3.42 | 3.17e-06 |
| 60 | 1.13 | 5.23e-01 | 2.99 | 9.69e-06 | 3.58 | 2.09e-03 | 3.49 | 1.15e-02 | 3.64 | 1.12e-03 | 3.44 | 3.11e-06 |
| 61 | 1.08 | 5.35e-01 | 3.01 | 9.62e-06 | 3.61 | 2.06e-03 | 3.62 | 1.13e-02 | 3.64 | 1.16e-03 | 3.49 | 3.00e-06 |
| 62 | 1.04 | 5.44e-01 | 3.01 | 9.59e-06 | 3.63 | 2.05e-03 | 3.54 | 1.15e-02 | 3.65 | 1.16e-03 | 3.49 | 3.00e-06 |
| 63 | 0.99 | 5.54e-01 | 3.03 | 9.53e-06 | 3.62 | 2.07e-03 | 3.70 | 1.13e-02 | 3.66 | 1.15e-03 | 3.51 | 2.98e-06 |
| 64 | 0.89 | 5.76e-01 | 3.03 | 9.50e-06 | 3.61 | 2.11e-03 | 3.70 | 1.13e-02 | 3.67 | 1.13e-03 | 3.50 | 3.13e-06 |
| 65 | 0.79 | 5.98e-01 | 3.04 | 9.45e-06 | 3.59 | 2.20e-03 | 3.70 | 1.13e-02 | 3.68 | 1.12e-03 | 3.50 | 3.30e-06 |
| 66 | 0.67 | 6.23e-01 | 3.06 | 9.37e-06 | 3.59 | 2.22e-03 | 3.60 | 1.16e-02 | 3.70 | 1.10e-03 | 3.52 | 3.30e-06 |
| 67 | 0.58 | 6.44e-01 | 3.07 | 9.32e-06 | 3.56 | 2.29e-03 | 3.60 | 1.16e-02 | 3.71 | 1.09e-03 | 3.63 | 2.99e-06 |
| 68 | 0.51 | 6.59e-01 | 3.08 | 9.30e-06 | 3.59 | 2.27e-03 | 3.69 | 1.18e-02 | 3.72 | 1.07e-03 | 3.65 | 2.92e-06 |
| 69 | 0.45 | 6.71e-01 | 3.09 | 9.25e-06 | 3.59 | 2.27e-03 | 3.69 | 1.18e-02 | 3.74 | 1.05e-03 | 3.69 | 2.81e-06 |
| 70 | 0.42 | 6.78e-01 | 3.11 | 9.36e-06 | 3.61 | 2.24e-03 | 4.95 | 1.02e-02 | 3.75 | 1.03e-03 | 3.70 | 2.79e-06 |
| 71 | 0.44 | 6.75e-01 | 3.12 | 9.26e-06 | 3.65 | 2.21e-03 | 4.98 | 1.02e-02 | 3.77 | 1.01e-03 | 3.75 | 2.65e-06 |
| 72 | 0.71 | 6.31e-01 | 3.13 | 9.22e-06 | 3.70 | 2.17e-03 | 4.98 | 1.02e-02 | 3.78 | 9.94e-04 | 3.76 | 2.61e-06 |
| 73 | 0.73 | 6.28e-01 | 3.15 | 9.27e-06 | 3.71 | 2.16e-03 | 4.80 | 1.07e-02 | 3.79 | 9.77e-04 | 3.76 | 2.88e-06 |
| 74 | 0.73 | 6.28e-01 | 3.17 | 9.13e-06 | 3.71 | 2.19e-03 | 5.00 | 1.05e-02 | 3.81 | 9.62e-04 | 3.75 | 3.06e-06 |
| 75 | 0.73 | 6.28e-01 | 3.20 | 8.98e-06 | 3.66 | 2.33e-03 | 4.76 | 1.11e-02 | 3.82 | 9.44e-04 | 3.76 | 3.13e-06 |
| 76 | 0.73 | 6.28e-01 | 3.24 | 8.70e-06 | 3.74 | 2.25e-03 | 4.76 | 1.11e-02 | 3.83 | 9.29e-04 | 3.78 | 3.08e-06 |
| 77 | 0.73 | 6.28e-01 | 3.25 | 8.66e-06 | 3.78 | 2.21e-03 | 4.70 | 1.12e-02 | 3.85 | 9.04e-04 | 3.81 | 3.01e-06 |
| 78 | 0.83 | 6.12e-01 | 3.25 | 8.76e-06 | 3.83 | 2.16e-03 | 4.70 | 1.13e-02 | 3.86 | 8.89e-04 | 3.84 | 2.96e-06 |
| 79 | 1.08 | 5.73e-01 | 3.28 | 8.63e-06 | 3.87 | 2.12e-03 | 4.70 | 1.13e-02 | 3.88 | 8.70e-04 | 3.84 | 2.96e-06 |
| 80 | 1.17 | 5.59e-01 | 3.29 | 8.63e-06 | 3.91 | 2.08e-03 | 4.73 | 1.12e-02 | 3.89 | 8.49e-04 | 3.84 | 2.94e-06 |
| 81 | 1.17 | 5.59e-01 | 3.29 | 8.90e-06 | 3.94 | 2.06e-03 | 4.47 | 1.19e-02 | 3.91 | 8.29e-04 | 3.86 | 2.91e-06 |
| 82 | 1.17 | 5.59e-01 | 3.31 | 8.96e-06 | 3.95 | 2.04e-03 | 4.47 | 1.19e-02 | 3.91 | 8.80e-04 | 3.89 | 2.85e-06 |
| 83 | 1.22 | 5.51e-01 | 3.33 | 8.84e-06 | 3.97 | 2.03e-03 | 4.22 | 1.24e-02 | 3.91 | 9.37e-04 | 3.91 | 2.78e-06 |
| 84 | 1.23 | 5.49e-01 | 3.34 | 8.74e-06 | 4.00 | 1.99e-03 | 4.37 | 1.22e-02 | 3.91 | 9.99e-04 | 3.92 | 2.76e-06 |
| 85 | 1.31 | 5.36e-01 | 3.36 | 8.68e-06 | 4.01 | 2.00e-03 | 4.69 | 1.19e-02 | 3.91 | 1.04e-03 | 3.94 | 2.72e-06 |
| 86 | 1.46 | 5.13e-01 | 3.40 | 8.43e-06 | 4.02 | 1.99e-03 | 4.69 | 1.19e-02 | 3.91 | 1.10e-03 | 3.95 | 2.70e-06 |
| 87 | 1.61 | 4.88e-01 | 3.40 | 8.94e-06 | 3.98 | 2.09e-03 | 4.62 | 1.21e-02 | 3.91 | 1.15e-03 | 3.96 | 2.67e-06 |
| 88 | 1.79 | 4.60e-01 | 3.40 | 9.10e-06 | 4.01 | 2.07e-03 | 5.22 | 1.14e-02 | 3.91 | 1.23e-03 | 3.99 | 2.60e-06 |
| 89 | 1.91 | 4.41e-01 | 3.40 | 9.29e-06 | 4.08 | 1.99e-03 | 5.22 | 1.14e-02 | 3.91 | 1.29e-03 | 4.05 | 2.47e-06 |
| 90 | 1.95 | 4.35e-01 | 3.40 | 9.75e-06 | 4.11 | 1.98e-03 | 5.94 | 1.06e-02 | 3.92 | 1.34e-03 | 4.13 | 2.30e-06 |
| 91 | 1.83 | 4.60e-01 | 3.49 | 9.17e-06 | 4.11 | 2.00e-03 | 5.45 | 1.18e-02 | 3.92 | 1.43e-03 | 4.17 | 2.21e-06 |
| 92 | 1.75 | 4.81e-01 | 3.52 | 9.04e-06 | 4.08 | 2.07e-03 | 5.45 | 1.18e-02 | 3.92 | 1.52e-03 | 4.19 | 2.16e-06 |
| 93 | 1.71 | 4.91e-01 | 3.57 | 8.73e-06 | 4.18 | 1.97e-03 | 5.49 | 1.18e-02 | 3.93 | 1.62e-03 | 4.20 | 2.15e-06 |
| 94 | 1.68 | 4.96e-01 | 3.63 | 8.40e-06 | 4.28 | 1.87e-03 | 5.59 | 1.17e-02 | 3.92 | 1.74e-03 | 4.20 | 2.18e-06 |
| 95 | 1.61 | 5.12e-01 | 3.66 | 8.21e-06 | 4.33 | 1.83e-03 | 5.59 | 1.17e-02 | 3.93 | 1.79e-03 | 4.19 | 2.36e-06 |
| 96 | 1.49 | 5.39e-01 | 3.68 | 8.35e-06 | 4.34 | 1.82e-03 | 7.68 | 9.39e-03 | 4.02 | 1.68e-03 | 4.28 | 2.13e-06 |
| 97 | 1.38 | 5.63e-01 | 3.68 | 8.71e-06 | 4.39 | 1.76e-03 | 8.22 | 8.81e-03 | 4.04 | 1.77e-03 | 4.34 | 1.98e-06 |
| 98 | 0.76 | 7.04e-01 | 3.84 | 7.66e-06 | 4.53 | 1.61e-03 | 8.22 | 8.81e-03 | 4.09 | 1.79e-03 | 4.42 | 1.76e-06 |
| 99 | 0.44 | 7.74e-01 | 3.93 | 7.05e-06 | 4.57 | 1.61e-03 | 10.60 | 6.23e-03 | 4.12 | 1.97e-03 | 4.51 | 1.52e-06 |
