# Supplementary material for: Go big or … don't? A field-based diet evaluation of freshwater piscivore and prey fish size relationships
Source: PLoS One. 2018 Mar 15;13(3):e0194092. doi: 10.1371/journal.pone.0194092 (PMC5854328; doi:10.1371/journal.pone.0194092)
Supplement: S3 Appendix — Table A: Review of gape-limit and maximum ingestible prey lengths estimates from the literature for our study piscivores. Fig A: Predator-specific maximum ingestible prey length. (DOCX) [file pone.0194092.s003.docx]

**S3 Appendix. Relative IP_max_ literature review.**

**Table A**. **Review of gape-limit and maximum ingestible prey lengths estimates from the literature for our study piscivores.** Studies are classified as either field surveys or studies measuring predator gape. The estimate is classified as a continuous maximum model, a single maximum value (100th percentile), or a 90th percentile value. a – p corresponds with literature derived estimates shown in Fig. A in S3 Appendix..

|  | Predator taxon | Prey taxon | Study Type | estimate Type | % Body Length | Reference |
| --- | --- | --- | --- | --- | --- | --- |
| a | Muskellunge | All prey | Field survey | Max. value | 47% | Bozek et al. (1999) |
| b | Muskellunge | Gizzard Shad | Field survey | Max. model | 12-28% | Wahl and Stein (1993) |
| c | Muskellunge | Bluegill | Field survey | Max. model | 13-20% | Wahl and Stein (1993) |
| d | Northern Pike | Gizzard Shad | Field survey | Max. model | 40-42% | Wahl and Stein (1993) |
| e | Northern Pike | Bluegill | Field survey | Max. model | 20-23% | Wahl and Stein (1993) |
| f | Walleye | All Prey | Field survey | Max. model | 24-51% | Knight et al. (1984) |
| g | Walleye | All Prey | Field survey | Max. model | 37-43% | Parsons (1971) |
| h | Walleye | Cyprinidae | Field survey | Max. model | 27-36% | Zimmerman (1999) |
| i | Largemouth Bass | Bluegill | Gape-limit | Max. model | 34-35% | Lawrence (1958) |
| j | Largemouth Bass | Gizzard Shad | Gape-limit | Max. model | 34-49% | Lawrence (1958) |
| k | Largemouth Bass | Largemouth Bass | Gape-limit | Max. model | 44-58% | Lawrence (1958) |
| l | Largemouth Bass | All Prey | Field survey | Max. model | 30-35% | Goldstein (1993) |
| m | Smallmouth Bass | Cyprinidae | Field survey | Max. model | 51-61% | Zimmerman (1999) |
| n | Smallmouth Bass | Cottidae | Field survey | Max. model | 38-53% | Zimmerman (1999) |
| o | Smallmouth Bass | Salmonidae | Field survey | Max. model | 32-51% | Zimmerman (1999) |
| p | Crappie | All Prey | Field survey | 90^th^ %tile value | 32% | Pierce et al. (2001) |

**Fig A**


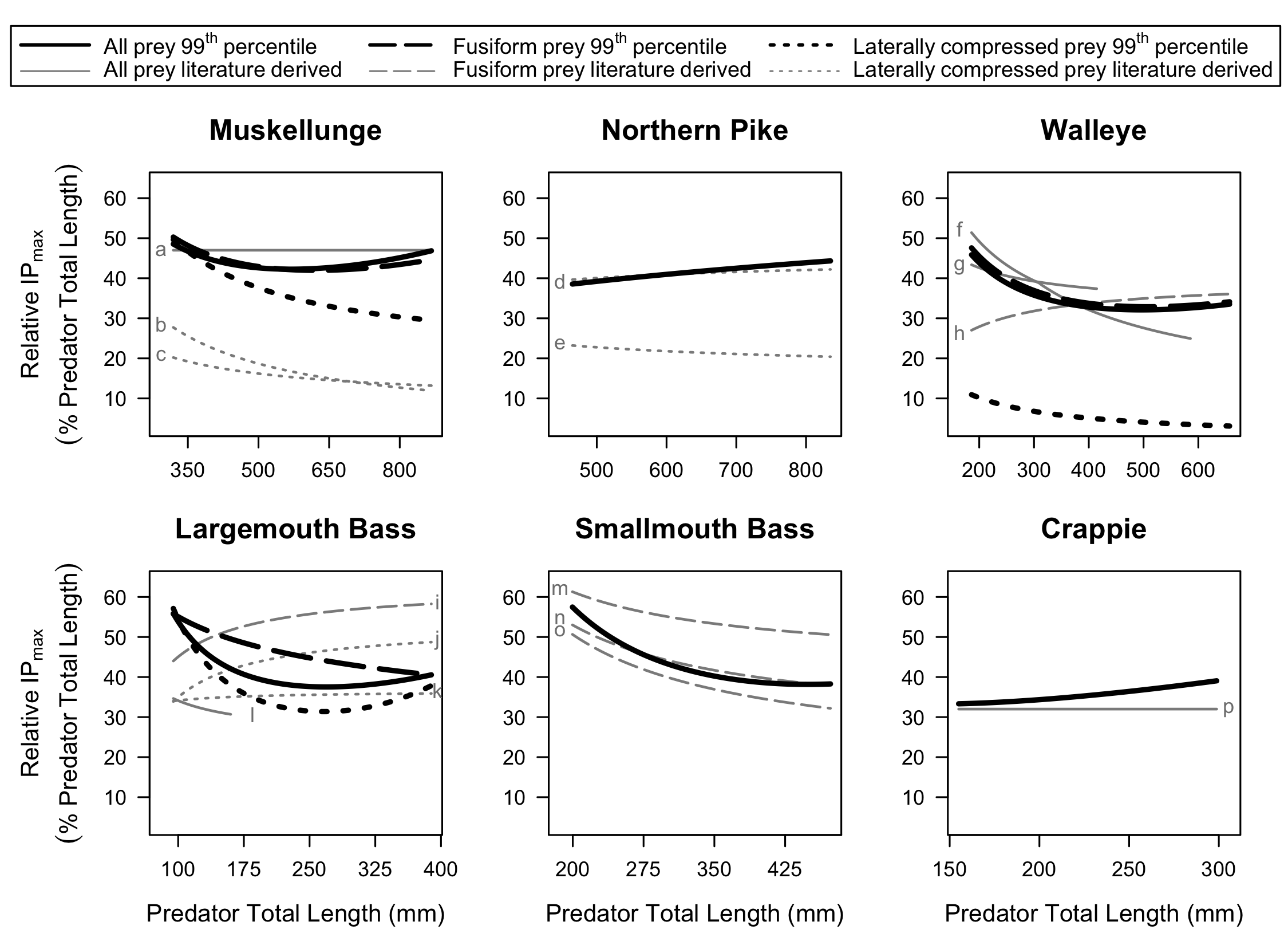


**Fig A. Predator-specific maximum ingestible prey length.**

Predator-specific maximum ingestible prey length (99^th^ percentile regression) shown as a percentage of predator total length (mm) for across predator total length (mm) for muskellunge (*Esox masquinongy*), northern pike (*Esox lucius*), walleye (*Sander vitreus*), largemouth bass (*Micropterus salmoides*), smallmouth bass (*Micropterus dolomieu*), and a grouped ‘crappie’ category (*P. nigromaculatus* and *P. annularis*). Estimates derived from this study are shown in black and estimates derived from the literature shown in gray. When applicable, we estimated maximum ingestible prey length for different prey body shapes: fusiform (dashed lines) and laterally compressed (dotted lines). Literature derived data are detailed in Table A in S3 Appendix.
