# Supplementary material for: Go big or … don't? A field-based diet evaluation of freshwater piscivore and prey fish size relationships
Source: PLoS One. 2018 Mar 15;13(3):e0194092. doi: 10.1371/journal.pone.0194092 (PMC5854328; doi:10.1371/journal.pone.0194092)
Supplement: S4 Appendix — Fig A. Rock bass percentile regression evaluation. (DOCX) [file pone.0194092.s004.docx]

**S4 Appendix. Rock bass percentile regression evaluation.**

**
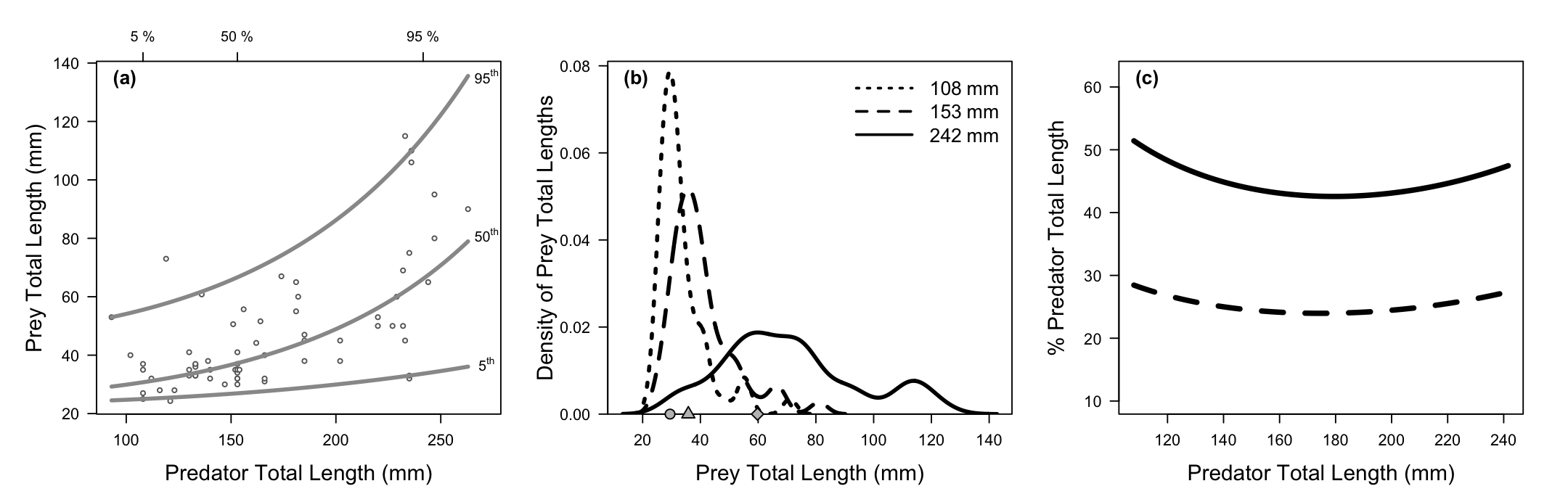
**

**Fig A. Rock bass percentile regression evaluation.** Rock bass (*Ambloplites rupestris*) (a) predator and prey fish total lengths (mm, n = 67) with 5^th^, 50^th^, and 95^th^ percentile regressions in gray lines; (b) kernel density distributions of model predicted prey total lengths (mm) for the 5^th^ (dotted line), 50^th^ (dashed line), and 95^th^ (solid line) percentile of predator total length (mm), which correspond to the top axis in (a); and (c) predator-specific maximum ingestible prey length (IP_max_; 95^th^ percentile regression; solid line) and central tendancy (IP_50_; 50^th^ percentile regression; dashed line) across predator total length (mm) shown as a percentage of predator total length. The 95^th^ is shown instead of the 99^th^ as in Fig 2 due to low sample size.
